# Programmable Deployment of Multistable Origami Metasurfaces through Algorithmically Distributed State Alteration

**DOI:** 10.34133/research.1287

**Published:** 2026-08-18

**Authors:** Soumyadeep Mondal, Tanmoy Mukhopadhyay, Susmita Naskar

**Affiliations:** School of Engineering, University of Southampton, Southampton, UK.

## Abstract

Achieving a prescribed 1- or 2-dimensional curvature from a flat shape, and their active modulation through far-field intelligent control, is crucial for a range of technologically demanding applications. In this article, we endeavor to exploit the bistable mechanics of waterbomb origami units, their strategic assembly, and spatially sequential alteration of local stability states to develop a new class of active metasurfaces. Based on a series of computational and experimental investigations, we propose a sequentially programmable approach of far-field stimuli-responsive activation, exploiting locally controllable bistability for achieving target curvatures that can hold their shapes without any external energy supply. A magnetic beam amplification system is developed and tested, demonstrating contactless stability state inversion of waterbomb units, which would essentially lead to prescribed curvatures in an origami-architected 1- or 2-dimensional domain. We further demonstrate that algorithmic control of localized stability states and their dynamic inversion can lead to robotic locomotion. The presented concept of spatially sequential stability state alteration, leading to the multifunctionality of actively reconfigurable metasurfaces, gripper function, and robotic locomotion, will find critical engineering applications in the fields of active and reconfigurable antennas, solar panels, aerodynamically adaptive aerofoils, soft robotics, energy-efficient grippers, biomedical apparatus, and programmable optical and acoustic devices.

## Introduction

Origami (from Japanese *ori*, meaning “to fold”, and *kami*, meaning “paper”) has been used as a documented art form since the 15th century, but was likely to have been used much earlier, as paper was introduced to Japan in the mid-7th century. Only in the mid-20th century has origami been used for the engineering purposes, and Koryo Miura, a Japanese astrophysicist, is considered the first developer of origami patterns for use in engineering [1]. He developed the Miura-Ori fold, which has been heavily utilized as foldable structures like solar panels for spacecraft and airbag expansion components [2,3]. Typically, origami research for engineering is split into 3 categories: expandable structures, complex origami shapes, and rigid lightweight structures. The nature of foldable patterns allows for highly expandable structures, which are essential in many critical environments where space is at a premium. For example, launch mass and volume are very limited for space applications, so having a mechanism that allows for a large surface area to be compacted into a very small volume for transit is very beneficial. It is for this reason that a large portion of research on engineering origami is focused on space applications, mainly deployable antennae and solar arrays [4–6]. Origami can be used to create highly complex shapes, which facilitates the creation of elaborate structures. For example, some car interior pieces on high-end performance cars are created from molds created using origami shapes [7]. Carbon fiber parts are often manufactured using special molds that can be produced from folding [8]. Folding can weaken a structure; however, when done correctly using specific folds, it can strengthen the structure beyond the material’s inherent capabilities, while maintaining very low weight compared to solid structures. In some cases, it even reveals highly desirable mechanical properties including improved specific strength and energy absorption capability due to control in failure path, along with programmable specific stiffness and Poisson’s ratios, leading to applications as a core of sandwich panels, vibration modulation, energy harvesting, and control devices [9–19]. The central theme of this paper is, however, on exploiting multistability [20–22] of strategically arranged bistable origami units to achieve targeted stable curvatures in metasurfaces. A metasurface, in the current context of shape and curvature modulation, is defined as a thin engineered surface composed of many small, designed units (often referred to as “unit cell” in the metamaterials literature [23–30]) whose local mechanical or geometric response can be programmed so that, as a whole, the surface can realize a target shape or curvature distribution that would not arise from a uniform conventional material. In other words, by varying the properties or actuation of these subunits across the surface, one can prescribe how the surface bends, stretches, or morphs in space, effectively turning it into a programmable, spatially tailored geometry rather than a passive sheet.

**Fig. 1.**
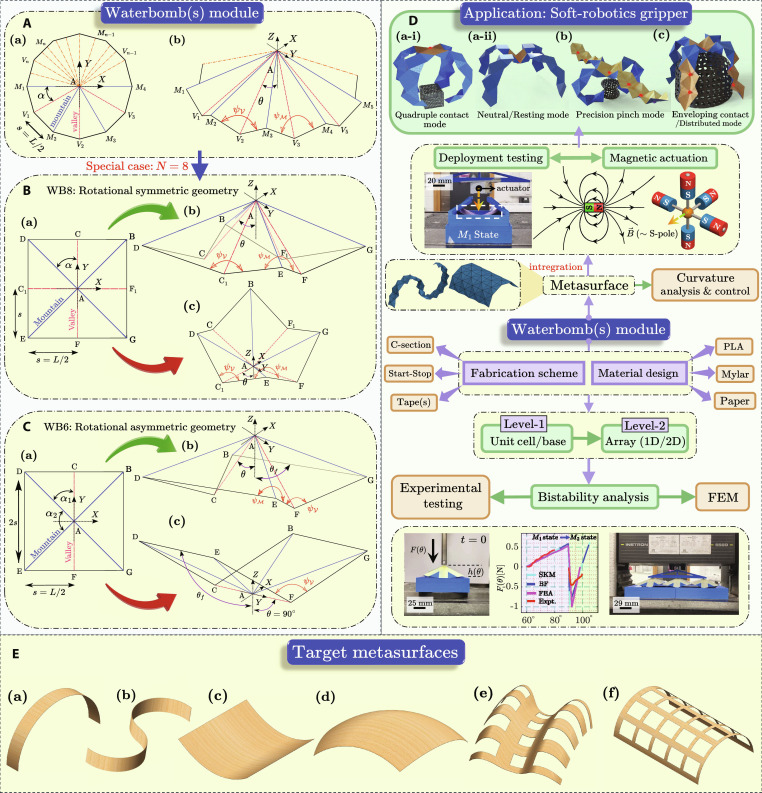
Conceptualization of multistable origami metasurfaces based on bistable waterbomb units. The design cycle for the metasurface begins with the basic origami building block (waterbomb module), which is extended to 1- and 2-dimensional functional assemblies including the introduction of active components for on-demand programmability. (A) A generic N-fold symmetric waterbomb (WB-N) unit. (B) A special case of 8-crease symmetric waterbomb (WB8) when N=8. (C) Typical representation of a rotationally asymmetric 6-crease waterbomb unit, WB6 (note that it cannot be obtained from the generic WB-N module as a special case). (D) An overview of the exploitation of WB6 and WB8 modules for developing 1D and 2D metasurfaces and soft-robotic grippers (including contactless magnetic actuation) through a combined numerical and experimental investigation. (E) Effective metasurface curvatures (tessellation of multiple waterbomb units in a plane) obtained through strategic assignment of stability states of the constituting waterbomb units locally. For the 2D curvatures, (c and d) primary and (e and f) secondary grid-like assemblies are proposed.

Based on folding’s geometric nature, properties, and applications, some common fold patterns in origami include Miura-ori, Waterbomb, Yoshimura, Kresling, Flasher, Blockfold, Eggbox, Ron-Resch, and Barreto–Mars, among others [31–34]. Origami waterbomb (WB) units are highly versatile fold patterns that developed concurrent to other origami bases as the art form evolved, especially in various applications on smart materials and robotics. Modern forms of origami utilize WB bases regularly, due to the symmetrical, simplistic, and modular design. The most commonly used variants of WB are the 6-crease WB (WB6) and 8-crease WB (WB8) variants, which consist of the same 2 diagonal folds and an additional 1 or 2 folds (as shown in Fig. 1B and C). Some well-known geometric properties of WB units include developability, rigid foldability, flat foldability, and bistability [31]. Developability means the ability of the WB to be developed from folding a flat sheet. Flat foldability indicates that WB can be fully folded into another flat configuration (assuming zero sheet thickness). WB6 exemplify such flat foldability nature. Rigid foldability, on the other hand, highlights the fact that WB units can be folded without inducing any deformation of its constituting facets, and the (elastic) deformations are only concentrated along the hinge-like creases (folds). For mathematicians, WB can be used to create a multitude of polyhedral shapes, including the cube, octahedron, and dodecahedron [35]. For engineers, WB has one main desirable property, it exhibits multistability (mostly bistable) behavior, meaning it has 2 stable states that it can switch between, given an external stimulus. Both the 6- and 8-crease WB have 4 natural states. Since the first state is determined by the initial folds, the WB has only 2 possible states, each sitting at an angle where the creases are at the minimum rotational displacement away from their set stable angles. The rigid-foldability characteristic of WB enables the use of spherical trigonometry to model its kinematics effectively. For instance, earlier, researchers [36,37] introduced a spherical trigonometry-based bistable kinematic model for the generalized (N-fold) WB and split-fold WB units. These WB units mostly conform to the rotationally symmetrical nature (i.e., equal sector angles). Chen et al. [38] have presented a comprehensive kinematic model for another widely popular 6-degree WB unit cell and its tessellations, where the zero-thickness facets as well as thick facets are discussed. They restricted the discussion up to a local symmetrical folding mode, where 4 mountain creases share the same dihedral angle, and 2 valley creases share a different but identical dihedral angle. Rodrigues et al. [39–41] later relaxed this symmetric restriction by varying dihedral angles and presented the kinematic and dynamic models. Zhang et al. [42] introduced a stability design approach based on the Euler–Lagrange energy functional, addressing the bifurcation motions of both 8-crease and 6-crease WB bases. Pratapa and Bellamkonda [43] analyzed the folding kinematics and deployability characteristics of 1-dimensional (1D) tessellated array of 6-crease WB bases (also termed Yoshimura base) accounting for the thickness of the facets (thin and thick). Mukhopadhyay et al. [44] introduced a tubular metamaterial whose microstructure is inspired by the 6-crease WB bases and uncovered a unique programmable deformation-dependent stiffness and shape modulation characteristics. A similar study on the folding mechanics of tubular WB-based metamaterial can be found in Ref. [45]. Grasinger et al. [46] presented an extensive multistability analysis of the symmetric 8-fold WBs. They demonstrated the existence of tristability in WB8 by introducing the concept of pretensioned torsional springs and also touched the mechanical aspect of quasi-1D arrays of such bases. Feng et al. [28] investigated the bistability of the flexible single-vertex origami bases (such as arbitrary WB and Miura unit cells) utilizing the conical shell model and the discrete creased model.

Previous research has covered many different areas and potential uses of origami, from flat and tubular programmable structures to multistability. However, much of the research focuses in the analysis of single base units including their perfectly periodic tessellation and deformation behavior, while very little research has been conducted on the uses of strategically assembled 1D or 2-dimensional (2D) arrays and target curvature attainment. Additionally, real-time actuation (actuation during motion) is relatively underinvestigated (more so for multistable origami structures that can hold its stable states at multiple configurations) as the focus has been on understanding the motion kinematics. For instance, origami-based soft robotics and active metasurfaces require real-time and controlled modulation of origami shape based on external actuation [47–52]. The soft robots can be useful for a wide range of stationary applications such as manipulation, gripping, manufacturing, safety, and healthcare [31,53,54]. Multimodal locomotion and navigation such as crawling, swimming, and grasping are also possible by using origami structural concepts [10]. Reconfigurable metasurfaces are crucial for applications like active antennas, morphing aerofoils and solar panels. Recently, Sharma et al. [55] proposed graded derivatives of Miura-ori architectures, leading to the possibility of a wide range of symmetric and asymmetric 1D curvatures. Such curvatures and deformed shapes do not possess multistability; thus, to hold a particular curvature, there needs to be continuous actuation. In the current work, we will focus on multistable architectures that can retain shapes at different stability configurations.

Mechanical or stimuli-responsive (such as magnetic, electrical, thermal, optical, pneumatic, chemical, etc. [32,56–65]) external actuation is a key component for lattice and origami-inspired soft robotics, grippers, and shape-changing metasurfaces. In this paragraph, we briefly review the actuation methods adopted in the literature concerning metamaterials and metasurfaces (including soft robotics) to understand their advantages and disadvantages [32]. Pneumatic actuation is widely used in soft robots for gripping because of their ability to handle delicate and small objects with on-and-off control [66]. Pneumatic actuation-based designs, however, may cause slow actuation and rupture failures due to the high strains required for actuation [50]. Vacuum actuation, which is similar to pneumatic actuation except that the actuation is performed using negative pressure, was exploited by Tawk et al. [67] to develop a linear actuator. The advantage of vacuum actuation over pneumatic actuation is that the actuators rely on negative pressure that prevents high stress from building up upon activation. They are also advantageous because vacuum actuators contract when activated, which is ideal for applications with space constraints. Li et al. [68] experimentally studied task-specific tunability of a vacuum-actuated soft robotic gripper made of an origami magic ball with a thin flexible membrane. Melancon et al. [69] utilized the Kresling pattern as a foundational element to realize inflatable tubular structures capable of switching between multiple deformation modes, while being globally actuated using a single air-pressure input. Lee et al. [70] introduced a deformable wheel robot inspired by an origami design, utilizing a WB origami pattern for its faces. The structure was constructed with polyethylene terephthalate film, while the fold lines incorporated fabric. The folding mechanism operated via a straightforward pulley system, which comprised a Kevlar wire encased in a Teflon tube and coupled with a coil spring. Xiang et al. [71] designed a continuum manipulator and soft gripper with a cable-driven actuator. This actuator works by retracting a cable that is embedded in strategic points in the structure. Eight cables were used to control the movement of the continuum manipulator, while one cable controls the grabbing motion of the soft gripper. This method managed to demonstrate the ability to cover a large range of motion by using the coupling effect of 2 actuation sections. However, the stability of the overall actuator during movement remains a challenge. Treml et al. [72] presented humidity-responsive actuators, integrated with the WB-based origami model, enabling autonomous sensing and transduction of an environmental signal into mechanical signal. Using magnetic actuation methods shows significantbenefits over other actuation methods because a magnetic field can penetrate through a wide range of materials and it is a contactless mechanism. The actuation time compared to other actuation methods is also relatively fast [73]. This means that a noncontact actuation method can be achieved without a delay in signal transmission. Noncontact actuation also means that the actual robot can opt to not have the actuator built in, which in turn reduces the weight. Soft actuators, inspired by animal muscle movement, are suited to drive a wide range of soft robots. One method for developing a soft actuator is by integrating magnetically responsive soft materials that can change their dimensions and, hence, stiffness when subjected to a magnetic field. These composite materials, also known as magnetic elastomers (MEs) are created by mixing micro- or nanosized magnetic materials into the elastomers [74,75] and gels [76]. Kitano et al. [77] utilized MEs and cable-driven actuators, combined with an electromagnet to demonstrate the basic concept of a manipulator. Electromagnets were used to create an attractive force, which increased the stiffness of the joint of the manipulator. The cables buried in the manipulator could only be pulled if the joint is not subjected to the attractive force. Coordinating the electromagnets and cable allowed the manipulator to bend toward a desired direction. Boyvat et al. [47] took a different approach to magnetically controlling their robots based on electromagnetic transmission, which was the same technology as wireless charging in mobile phones. Power was transmitted from an external coil to the internal coil via a magnetic field. This power received by the internal coil was then used to generate the current required for heat generation that would deform the shape memory alloy used to construct their robot. By deforming individual shape memory alloy, a specific movement could then be produced accordingly. Bowen et al. [78,79] provided a magnetically actuated dynamic model for the self-folding behavior of origami unit (8-crease WB and Shafer’s Frog Tongue), where magnetic material (magnetoactive elastomer and permanent magnets) were patched onto the rigid facets. The generated magnetic torque was used as the primary actuating factor to manipulate the joint rotations of origami bases. One limitation of this model arises from the use of multiple magnets on each facet. The increased overall mass and the complex interactions between magnetic fields can lead to deviations from the expected precise actuation modes. Cui et al. [80] recently proposed a modified (magnetic) bar-hinge model to analyze a similar origami structure, where panels (facet) are made as the assembly of a soft substrate layer and a soft magnetically actuated unit layer. They demonstrated the proposed model by applying it to 2 different case studies (6-crease WB pattern and a soft gripper, made of square and triangular panels). In this work, we will primarily focus on proposing magnetic actuation in the present context of multistable metasurfaces, leading to localized control and dynamic inversion of stability states. However, it is worth mentioning that other actuation methods can also be implemented for localized stability state inversion, and the main contribution of this work lies in proposing the concept of sequential and algorithmically distributed state alteration for achieving active curvature reconfigurability rather than developing a new actuation strategy.

A careful review of the literature concerning origami metamaterials and metasurfaces reveals that despite extensive research on the mechanical behavior, kinematics, and stability of individual WB origami units, much of the existing literature remains focused on unit-cell level characterization, their periodic assembly, or quasi-1D arrangements with limited capability of active and bidirectional curvature control [44,46,81–85]. Prior investigations have primarily addressed analytical modeling, stability conditions, and material implementations of single or isolated units and their perfectly periodic tessellations, often neglecting their assembly into spatially programmable arrays capable of achieving continuous and discrete curvature modulation [86–91]. The aspect of multistability in origami metasurfaces (i.e., an array of connected unit cells) that has the potential to hold prescribed shapes and curvatures along the motion path without continuous supply of power has not been adequately investigated. Considering an array of m×n WB units, with each of the unit possessing 2 stable states, there can be 2*^mn^* stable configurations of the metasurface, leading to a vast scope of curvature modulation along with their dynamic and sequential control [92–94] for achieving robotic motion [95]. The challenge of real-time actuation, especially in distributed and sequential stability state alteration, remains largely unaddressed in the context of multistable origami structures. In particular, the controlled transformation of global shape by locally altering bistable states across an array of units, without compromising rigidity or introducing structural complexity, has not been sufficiently explored. This opens a critical research opportunity in architecting programmable curvatures through coordinated dynamic local state transitions that has the potential for direct multifaceted functionalities like metasurfaces and gripper as well as robotic locomotion. The need for fast, contactless, and reversible actuation schemes further motivates the integration of magnetically responsive systems for activating localized stability state changes of constituting origami units. This study aims to address these gaps by proposing a novel design and deployment methodology for programmable origami metasurfaces based on algorithmically distributed stability state alteration, supported by a magnetic beam-based actuation strategy for real-time curvature control.

Achieving prescribed 1D or 2D effective curvatures in stable configuration (refer to the illustrative subfigures corresponding to metasurfaces in Fig. 1E) from a flat domain, and their active modulation through far-field intelligent control, is crucial for a range of engineering applications such as active and reconfigurable antennas, aerodynamically adaptive aerofoils, soft robotics, energy-efficient grippers, biomedical apparatus, and programmable optical and acoustic devices. In this article, we would exploit the bistable mechanics of WB origami units, their strategic assembly, and far-field stimuli-driven spatially sequential alteration of local stability states to develop a new class of active metasurfaces. A comprehensive overview of the current study concerning the constituting bistable WB units and their assemblies leading to actively controllable curvatures is illustrated in Fig. 1. The present paper is organized as follows. In the “Analytical modeling of elementary origami kinematics” section, we present the theoretical models of bistability based on 2 approaches: spherical trigonometry and bifurcation theory, beginning with a generalized *N*-fold WB configuration. Subsequently, we focus on the 8-crease WB as a specific example of the *N*-fold structure and examine an alternative geometry, the 6-crease WB (equivalent to a Yoshimura unit cell [43,96,97]; note that it is not achievable by N=6 in a rotationally symmetric WB configuration as discussed in the following sentences), as a starting point for further discussions. WB configurations are broadly categorized into 2 types based on the sector angles. Configurations with equal sector angles between adjacent creases are termed rotationally symmetric configurations. In Fig. 1A and B, the WB configuration has a sector angle of α between each crease. On the other hand, configurations with different sector angles are termed rotationally asymmetric WB configurations. For instance, Fig. 1C exemplifies a 6-crease WB with 2 sector angles, $\alpha_{1}$ and $\alpha_{2}$. In the “Conceptual assembly for metasurface” section, we explore the functional assembly and fabrication aspect of WB-based structures through digital design and physical modeling. Numerical and experimental demonstration of active origami metasurfaces is discussed thereafter, beginning with the evaluation of performance parameters at the unit WB cell level. Following this, experimental tests are presented and validated against numerical and theoretical models for various fabricated WB units and their assembled arrays. Finally, the feasibility of magnetic actuation is demonstrated by incorporating magnets in the WB structures. Considering a range of strategic assemblies, we demonstrate the curvature modulation in 1D and 2D arrays of WB units, leading to functionalities like reconfigurable metasurfaces, gripper functions and robotic locomotion. For appropriate contextualization of the presented work, conclusions and engineering perspectives are summarized at the end of this paper.

## Results and Discussion

### Analytical modeling of elementary origami kinematics

In this section, we have briefly presented the theoretical analysis at the level of single-vertex origami unit, before extending it onto the global tessellated surface level, which is physically tested and discussed in the later sections. This section is categorized into 2 parts, where first the WB units with rotationally symmetric geometry (equal sector angle of α) are discussed based on kinematic and energy-based methods. Afterwards, one instance of the WB units with different sector angles (i.e., 6-crease WB) is presented. Figure 1A to C show the schematics of the respective category of WBs with rotationally symmetric and nonsymmetric configurations. The standard sign convention of fold angles and dihedral angles is maintained throughout the study [98]. For instance, the fold angle (ϕ), which is the relative rotation between the 2 facets, is taken positive (or negative) for valley (or mountain) folds, whereas the dihedral angle (ψ) is π radian less the fold angle between the positive sides of the 2 facets. In both cases, the system is taken as one degree of freedom (dof). For instance, the angle (θ) between the negative Z-axis and the valley fold is assigned as the only input variable to control the motion responses of the system (refer to Fig. 1A to C). A few assumptions are maintained throughout the study, which can be summarized as follows: (a) The facets (faces) are taken as rigid (nonexistence of stretching or bending deformation) and they do not intersect with each other. (b) A single WB unit consists of one single developable vertex (the sum of all the sector angles, meeting at the central vertex is 2π), i.e., $\sum \;\alpha_{i}=2\pi$. (c) The WB remains intact throughout the motion, with initially connected facets staying joined.

*WBs with rotationally symmetric geometry.* To keep the discussion general, an *N*-fold WB unit ($N=\left\{\;k\in \mathbb{N}|k\geq 6\;\text{and}\;k\;\text{is}\;\mathrm{even}\;\right\}$) with $N_{M}=N/2$ mountain creases and $N_{V}=N/2$ valley creases is considered. Note that the mountain and valley creases alternate in the configuration. Considering Fig. 1A, the same category of crease (mountain or valley) retains exactly the same fold angles in all possible configurations of the origami unit throughout its motion, which can be written as follows:

$$\psi_{M_{1}}=\psi_{M_{2}}=\cdots =\psi_{M_{N_{M}}}=\psi_{M}$$ (1A)

$$\psi_{V_{1}}=\psi_{V_{2}}=\cdots =\psi_{V_{N_{V}}}=\psi_{V}$$ (1B)

The sector angle can be given as $\alpha =2\pi /\left(N_{V}+N_{M}\right)$ for all the crease folds. The stability analysis of such WB units can be done using the potential energy function of the whole unit, which can be given as follows:

$$U\left(\theta \right)=N_{M}\left[\frac{1}{2}k_{M}{\left(\psi_{M}\left(\theta \right)-\psi_{M}^{R}\right)}^{2}\right]+N_{V}\left[\frac{1}{2}k_{V}{\left(\psi_{V}\left(\theta \right)-\psi_{V}^{R}\right)}^{2}\right]$$ (2)

where $k_{M}$ and $k_{V}$ can be termed the torsional spring-equivalent crease stiffnesses for the mountain and valleys folds, respectively. Here, the dihedral angles $\psi_{M}$ and $\psi_{V}$, following the spherical kinematic based analysis of Hanna et al. [37], can be summarized as follows:

$$\psi_{M}\left(0\leq \theta \leq \pi -\alpha \right)=2\pi -{\text{cos}}^{-1}\left(1-2{\text{sin}}^{2}\theta \right)$$ (3A)

$$\begin{array}{c} \begin{array}{c} \begin{array}{ll} \psi_{V}\left(0\leq \theta <\pi /2\right)=2\pi & \;-2{\text{cos}}^{-1}\left[\text{cot}\alpha \text{tan}\frac{{\text{cos}}^{-1}\left(1-2{\text{sin}}^{2}\theta {\text{sin}}^{2}\alpha \right)}{2}\right]\\ & \;-2{\text{cos}}^{-1}\left[\text{cot}\theta \text{tan}\frac{{\text{cos}}^{-1}\left(1-2{\text{sin}}^{2}\theta {\text{sin}}^{2}\alpha \right)}{2}\right] \end{array} \end{array} \end{array}$$ (3B)

$$\begin{array}{c} \begin{array}{c} \begin{array}{ll} \psi_{V}\left(\pi /2<\theta \leq \pi -\alpha \right)\; & =2{\text{cos}}^{-1}\left[\frac{-{\text{sin}}^{2}\alpha \text{sin}2\theta }{\text{sin}\left({\text{cos}}^{-1}\left(1\;-\;2{\text{sin}}^{2}\theta {\text{sin}}^{2}\alpha \right)\right)}\right]\\ & -2{\text{cos}}^{-1}\left[\text{cot}\alpha \text{tan}\frac{{\text{cos}}^{-1}\left(1\;-\;2{\text{sin}}^{2}\theta {\text{sin}}^{2}\alpha \right)}{2}\right] \end{array} \end{array} \end{array}$$ (3C)

Note that each θ corresponds to a different folded configuration of the WB unit. It can be seen that the mountain angle, $\psi_{M}$, is independent of the number of crease, N. The $\psi_{M}^{R}=\psi_{M}^{R}\left(\theta_{0}\right)$ and $\psi_{V}^{R}=\psi_{V}^{R}\left(\theta_{0}\right)$ are the corresponding rest dihedral angles of initial stress-free configuration. Equation (3) highlights the dimension-independent nature of WB fold angles, although the potential energy in Eq. (2) does depend on the dimension of the specimen, such as the height of the vertex and the fold dimensions. As in the present study, we mostly consider the testing of 8-crease WB (WB-N: N=8) (refer to Fig. 1B)-based origami surfaces; the dihedral angles corresponding to its valleys can be calculated from Eqs. (3B) and (3C) by substituting N=8 into it; however, the same corresponding to the 4 mountains will be the same as Eq. (3A). Besides the approach discussed so far, a robust kinematic model considering the bifurcation (BF) motions for the 8-crease (square) WB can be established [42], where the total potential energy [$U\left(\theta \right)$] of the system is given as the combination of 2 potential energies corresponding to 2 distinct bifurcation states. This can be written as follows:

$$U\left(\theta \right)=\text{min}\left[U_{\mathrm{BF}1}\left(\theta \right),\;U_{\mathrm{BF}2}\left(\theta \right)\right]\;\forall \theta \in \left[0\;\pi \right]$$ (4)

Here, $U_{\mathrm{BF}1}$ and $U_{\mathrm{BF}2}$ are the potential energy functions corresponding to first and second bifurcation motions, respectively. The closed-form expressions of $U_{\mathrm{BF}1}$ and $U_{\mathrm{BF}2}$ are the same as Eq. (2); however, the dihedral angles ($\psi_{\;M},\psi_{V}$) corresponding to each bifurcation state are different from the earlier kinematic model. The closed form expressions of dihedral angles for both BFs are summarized in Table 1. According to Eq. (4), for each value of θ, the WB (WB8) will select the configuration with the minimum potential energy, among 2 possible bifurcated configurations. In the present study, for obtaining adequate confidence, both the aforementioned models are utilized to calculate the inverting forces and the comparison study with the experiments is given in the “Numerical and experimental demonstration of active origami metasurfaces” section.

**Table 1.** Dihedral angles ($\psi_{ℳ},\psi_{V}$) of WB8 concerning bifurcation

	$\psi_{\;M}$	$\psi_{V}$
Bifurcation motion - I	2θ	$2\pi -2{\text{cot}}^{-1}\left[\frac{\text{cos}\theta \left(\text{sin}\theta +\sqrt{2}\right)}{\text{sin}\theta -\sqrt{2}{\text{cos}}^{2}\theta }\right]$
Bifurcation motion - II	$2\left(\pi -\theta \right)$	$2{\text{cot}}^{-1}\left[\frac{\text{cos}\theta \left(\sqrt{2}-\text{sin}\theta \right)}{\text{sin}\theta +\sqrt{2}{\text{cos}}^{2}\theta }\right]$

*WBs without rotationally symmetric geometry.* Here, the WBs with 2 different sector angles ($\alpha_{1}$ and $\alpha_{2}$) are presented. A 6-crease WB can be exemplified in this category (refer to Fig. 1C). It is important to note that the term of 6-crease unit here is different from that of the 6-crease model (WB-N: N=6), which can be derived from an earlier symmetric *N*-fold WB unit (refer to the discussion on WBs with rotationally symmetric geometry). As this kind of nonrotationally symmetric 6-crease WB unit (WB6) is not included in the general set of symmetric WB-N, the theoretical models for such unit are highlighted in this section.

Based on the current WB without rotational symmetric geometry, $N_{M}=4$ and $N_{V}=2$, whereas the 2 sector angles can be written as $\alpha_{1}=\pi /N_{M}$ and $\alpha_{2}=\pi /N_{V}$. A 3D coordinate geometric approach is used to derive the dihedral angles of the creases in WB6 here. The derivation is given in Section S1 (refer to the Supplementary Materials) and the final explicit expressions for the angles are summarized here

$$\psi_{M}\left(0\leq \theta <\pi /2\right)=\pi +{\text{cos}}^{-1}\left(2\text{sin}\theta -1\right)$$ (5A)

$$\psi_{M}\left(\pi /2\leq \theta_{f}<\pi \right)=0$$ (5B)

$$\psi_{V}\left(0\leq \theta <\pi /2\right)={\text{cos}}^{-1}\left(\frac{1-3\text{sin}\theta }{1+\text{sin}\theta }\right)$$ (5C)

$$\psi_{V}\left(\pi /2<\theta_{f}\leq \pi \right)=2\left(\pi -\theta_{f}\right)$$ (5D)

It is important to note that the expressions here are derived based on 2 possible stable configurations shown in Fig. 1C, b and c. Also, note that it may seem that the shapes of the 2 stable states shown in Fig. 1C are preassumed and that the derivations are carried out based on them. However, this is not the case. The existence of these stable shapes can be conceived from the perspective of a bifurcation generally occur at the fully unfolded state ($\theta_{f}=\theta =\pi /2$) during its folding motion. The 2 stable shapes lie on distinct bifurcated motion paths, each corresponding to a local potential energy minimum of the associated path. This bifurcation perspective is discussed in the following paragraph and thus offers an advantage of this approach compared to conventional geometry-based kinematic analysis. The angle θ varies until it reaches the unstable flat configuration (θ=π/2); afterwards, its value becomes constant at π/2 for all the configurations beyond this unstable position. Now, to get the dihedral angles for all the configurations beyond this position, another input angle, $\theta_{f}$, needs to be used as the dof, which can be defined as the angle between the larger triangular facet (without valley) and the negative *Z*-axis. Note that the angle $\theta_{f}$ could have been used in the formulation for below this position as well instead of using θ; however, the θ is used here for the sake of simplicity in the formulation. It is needed to be clarified here that this WB is also a single dof and either θ or $\theta_{f}$ can be used as its dof. The expression of total potential energy, $U\left(\theta \vee \theta_{f}\right)$ based on this model, will be similar to that of Eq. (2).

A bifurcation-based kinematic model [42] for the present 6-crease WB is presented here, wherein the potential energy can be stated as the minimization of potential between 2 possible bifurcated motions (configurations).

$$U\left(\theta \vee \theta_{f}\right)=\text{min}\left[U_{\mathrm{BF}1}\left(\theta \vee \theta_{f}\right),\;U_{\mathrm{BF}2}\left(\theta \vee \theta_{f}\right)\right]\;\forall \theta_{f}\in \left[0\;\pi \right]$$ (6)

The dihedral angles ($\psi_{M},\psi_{V}$) corresponding to 2 bifurcation motions are summarized in Table 2.

**Table 2.** Dihedral angles ($\psi_{ℳ},\psi_{V}$) of WB6 concerning bifurcation

	$\psi_{M}$	$\psi_{V}$
Bifurcation motion - I	π	$2\left(\pi -\theta_{f}\right)$
Bifurcation motion - II	$\pi -\text{sgn}\left(\theta_{f}-\frac{\pi }{2}\right)\left(\pi -{\text{cos}}^{-1}\left[\frac{3{\text{cos}}^{2}\theta_{f}-1}{1+{\text{cos}}^{2}\theta_{f}}\right]\right)$	$2\theta_{f}$

*Remarks on crease stiffness and inverting force:* To simulate the folding of creases, we use a torsional spring concept replicating hinge joints. This concept of folding is similar to a method called a small-length flexural pivot [99]. Here, the crease regions are considered as an individual flexible (contrary to rigid assumption for facets) compliant structure that achieves movement or flexibility through the bending (flexure) of its material. Following this concept, the equivalent stiffness of the creases ($k_{M}$ and $k_{V}$) can be given as $\mathrm{EI}/L_{c}$, where EI is the flexural stiffness and $L_{c}$ is the length of the crease between 2 facets, joining at this crease. However, the crease stiffnesses in actual physical models largely depend on its actual geometry and material properties.

In the present study, the force required to invert the origami structure from one of its stable states ($M_{1}$) to another state ($M_{2}$) is measured by examining the potential energy stored in the system, $U\left(\theta \right)$, as the WB’s vertex is brought to its current stable position [defined as $z=\left(L/2\right)\text{cos}\theta$)] under the influence of an externally applied force, F, directed along the *Z*-axis. Consequently, the force $F\left(\theta \right)$ can be expressed as follows:

$$F\left(\theta \right)=-\frac{\mathrm{dU}\left(\theta \right)}{\mathrm{dz}}=-\frac{\mathrm{dU}\left(\theta \right)}{\mathrm{d\theta}}\frac{\mathrm{d\theta}}{\mathrm{dz}}=\frac{2}{L\text{sin}\theta }\frac{\mathrm{dU}\left(\theta \right)}{\mathrm{d\theta}}$$ (7)

where L is the side length of the WB (WB8 and WB6) (considered square-shaped in the current analysis). As the force (F) is a function of θ, it will be seen that it reaches maximum at the bifurcation point (θ=π/2) where inversion from $M_{1}$ to $M_{2}$ occurs through snapping. Therefore, the value of this critical inversion force can be calculated as $F\left(\theta =\pi /2\right)$. This value is crucial to know as it directly gives an idea of how much minimum force needs to be generated by the present external magnetic system (discussed later) to get contactless actuation for present origami units.

*Remarks on rest fold angles (*$\psi_{M}^{R},\psi_{V}^{R}$*) for the WB:* Here, we have presented a brief understanding concerning how the rest angles (which are normally taken as a known quantity, often measured based on the behavior of the physical sample) for the mountain and valley creases can be conceptualized qualitatively through analytical means. In real settings, if we are considering making creases from sheets or papers or films (which are generally used in the origami art), the sheet is folded along the crease line as per the design. In fact, Feng et al. [100] have beautifully highlighted 2 specific folding methods by means of compression and pure bending to form a crease on the plain sheet. Among these 2 methods, the compression-based forming process is common and widely adopted in the origami art. In this method, the sheet is first compressed between 2 rigid plates to the extent where the distance between 2 plates becomes close to double the thickness of the sheet and then the plates are released. After the release, residual plastic deformation at the crease location is retained, which forms the crease and keeps it sustained at a particular natural crease angle, which can be called rest angle in the present context. Such extremely localized plastic deformation in the crease line is realized in the second forming method as well. The extent of localized plastic deformation of the sheet material eventually decides the rest angle as well as the rotational stiffness of the crease. Furthermore, the flattening of this newly formed crease by some external influences (such as loading, weight, etc.) can make the crease sustained at a new rest angle, which essentially make rest angles folding history dependent [101]. However, for the sake of analyzing origami-inspired mechanical structures, we generally idealize the creases as individual torsional springs (with some nonzero rotational stiffnesses). Such an assumption can be made practical in origami behavior by designing the creases as hinge joints with added torsional springs within it. As torsional springs typically come with a specific free leg position (the angular orientation of the spring’s legs when it is in an unloaded state), set during the manufacturing process, the rest angle depends on this [102]. However, the effect of external influences (such as spring material fatigue or weight of the structure) on changing the initial rest angle cannot be overlooked in this case as well, for a more accurate analysis spanning across the service life.

Although the natural rest angles cannot be obtained directly due to their dependence on various parameters, such as those related to plastic deformation and manufacturing conditions, the effect of external parameters (e.g., the structure’s mass) on the preassumed rest angles ($\psi_{M}^{R}$ and $\psi_{V}^{R}$) of the origami structure can be addressed analytically. Such analytical models can be used for qualitative understanding and can be adopted with appropriate parameters tuning for more quantitative characterization. As both the rest angles are the function of the orientation angle θ, we are introducing another parameter $\theta_{0}^{p}$, which defines the initial natural rest value of θ in massless WB unit. The angle $\theta_{0}^{p}$ refers to the initial rest configuration right after release from plastic deformation in massless sheet-based origami, or the configuration of origami-inspired mechanical structure with torsional springs at their free leg positions. After the inclusion of mass effect, this initial rest angle will be saturated at another value (let us define it as $\theta_{0}^{\;f}$), which needs to be determined. To determine the same, the energy minimization with added gravitational potential energy term is utilized. For that, the location of mass center $C_{M}$ (refer to Fig. S2A and B) of the whole WB unit needs to be obtained as

$$R=\frac{\sum_{i=1}^{N}m_{i}r_{i}}{\sum_{i=1}^{N}m_{i}}$$ (8)

Here, $m_{i}$ denotes the mass of the individual facet. R and r denote the center of mass of the WB unit and its individual facet, respectively. N is the number of creases present in the unit, which is 8 for WB8 and 6 for WB6. As uniform and same mass ($m_{f}$) for each facet is considered (i.e., $m_{i}=m_{f}$), Eq. (8) can be further simplified as follows:

$$R=\frac{1}{N}\sum_{i=1}^{N}r_{i}$$ (9)

The mass center $r_{i}$ for the individual facet can be calculated from the coordinates of the triangular facet (refer to Fig. S2C) as follows:

$$r_{i}=\frac{1}{3}\left(\sum_{j=1}^{3}x_{j}^{i}\;\hat{i}+\sum_{j=1}^{3}y_{j}^{i}\;\hat{j}+\sum_{j=1}^{3}z_{j}^{i}\;\hat{k}\right)$$ (10)

Considering the coordinate system shown in Fig. S2, $\left(x_{1}^{i}\;y_{1}^{i}\;z_{1}^{i}\right)$ will be $\left(0\;0\;0\right)$. The exact expressions for $\left(x_{2}^{i}\;y_{2}^{i}\;z_{2}^{i}\right)$ and $\left(x_{3}^{i}\;y_{3}^{i}\;z_{3}^{i}\right)$ are provided in Section S2. The final closed-form expression of R for WB8 (refer to Fig. S2A) can be obtained as

$$\begin{array}{c} \begin{array}{c} R=-\left(\frac{\sqrt{2}}{6}\;L\text{cos}\left(\frac{\pi }{2}+\text{atan}\left(\frac{\text{cos}\theta \left(\text{sin}\theta -\sqrt{2}\right)}{\sqrt{2}{\text{cos}}^{2}\theta +\text{sin}\theta }\right)\right)+\frac{L\text{cos}\theta }{6}\right)\hat{k}=-\left(h-h_{\mathrm{COM}}\right)\hat{k} \end{array} \end{array}$$ (11)

The same for the WB6 unit (refer to Fig. S2B) can be derived as follows:

$$R=-\left(\frac{2\;L\;\text{cos}\;\theta_{f}}{9}+\frac{2\;L\;\text{cos}\;\theta_{f}}{9\;\left({\text{cos}}^{2}\;\theta_{f}+1\right)}\right)\hat{k}=-\left(h-h_{\mathrm{COM}}\right)\hat{k}$$ (12)

By calculating $h_{\mathrm{COM}}$ from the above expressions of R, the gravitational potential energy stored in the unit at a given folded configuration θ (as shown in Fig. S2A and B) can be given as $U_{g}={\mathrm{Nm}}_{f}{\mathrm{gh}}_{\mathrm{COM}}$, where g is the acceleration of gravity. Therefore, the total potential energy of the system will now be $U_{t}=U+U_{g}$ [as defined in Eq. (2)], which is then minimized numerically to determine the stable configurations. Among 2 stable points, the first one is taken as the new rest configuration, associated with the rest angle $\theta_{0}^{f}$, which can be termed the final rest angle.

In this context, if the magnetic setup is attached at the central vertex of the unit (as discussed in the “far-field magneto-active control of metasurface shape and curvature” paragraph below), the mass of the tip magnet will also influence the rest configuration. This can be taken into consideration by its associated potential energy as $U_{\mathrm{mag}}=m_{\mathrm{mag}}\mathrm{gh}$, where $m_{\mathrm{mag}}$ is the total mass of the magnet setup. It is above the flat configuration by h=0.5Lcosθ. Thereafter, by a similar way of energy minimization of the new total energy $U_{t}=U+U_{g}+U_{\mathrm{mag}}$, we can get rest configurations.

**Fig. 2.**
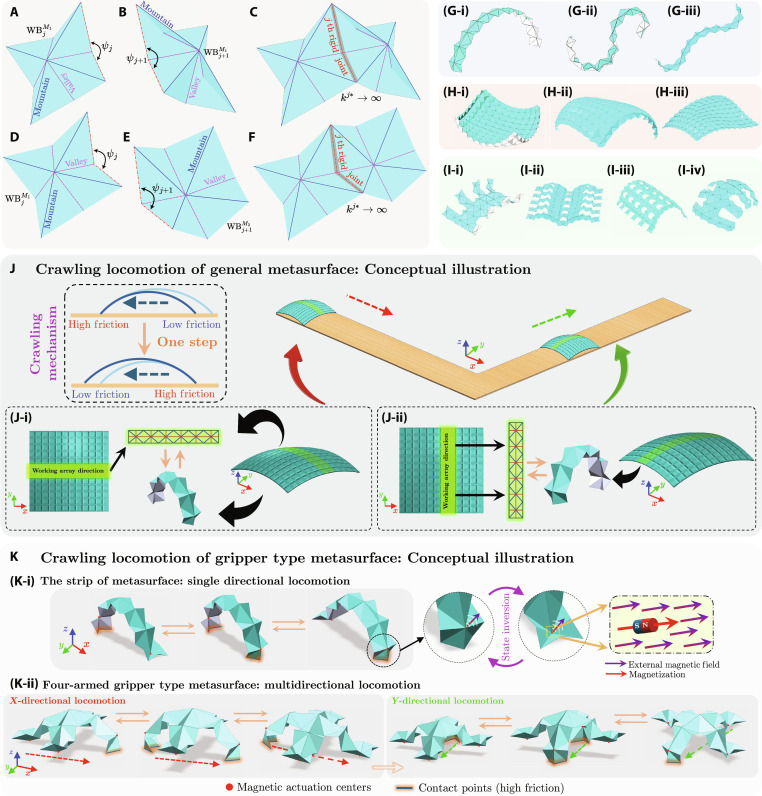
Strategic primary and secondary assembly of multiple waterbomb units for metasurfaces with multifaceted functionality. (A to F) Assembly of 2 waterbomb units (WB8) with different stability states. The first case considers 2 waterbomb modules, i.e., (A) *j*th and (B) (*j*+1)th WB, at their $M_{1}$ states. The second case considers 2 waterbomb modules i.e., (D) *j*th and (E) (*j*+1)th WB, at their $M_{1}$ and $M_{2}$ state, respectively. In both cases, 2 WB units are assembled at their *j*th joint (C and F). Here, the *j*th joint (crease connection) is considered to be rigid (thus, not an origami folding joint), which mathematically can be equivalent to a torsional spring with infinite spring stiffness. All the facets are assumed to be ideally rigid throughout the folding process. Such rigid-folding assumption and kinematic compatibility condition enforce the equality of the dihedral angles, $\psi_{j}$ and $\psi_{j+1}$ of *j*th and (*j*+1)th WB at their *j*th crease joint. The WB8 units can be assembled into metasurfaces to achieve various curvatures. (G) 1D array of units. (H) Various curvatures through primary metasurface assembly. (I) Various curvatures through secondary metasurface assembly. (J) Conceptual illustration of locomotion of a generic metasurface (formed using 1D or 2D array of multiple waterbomb units) exploiting frictional anisotropy and sequential periodic alteration of stability states locally. (K) Conceptual illustration of magnetically controlled single and multidirectional locomotion of a gripper-type architecture.

*Remarks on the effect of surrounding WB units:* As in the present paper, the WB units are extended to an equivalent surface level through geometric assembling and the bistable nature of individual units are exploited later to achieve effective curvature at the global level; the effect of neighboring WB units on the bistable behavior of an individual unit is discussed here. Let us consider one quasi-1D array of n number of identical WBs (WB8), attached along the edge (refer to Fig. 2C and F, where crease connection between 2 WB8 units are illustrated). Here, the configuration of individual units can be of either *M*_1_ state or *M*_2_ state. The overall potential energy ($U_{s}$) of such WB assembly can be given mathematically as follows:

$$U_{s}=\sum_{j=1}^{n}U^{j}+\sum_{j=1}^{n-1}U^{j*}$$ (13A)

$$=\sum_{j=1}^{n}U^{j}+\sum_{j=1}^{n-1}\left[\frac{1}{2}k^{j*}\left(\psi^{j}-\psi^{j+1}\right)\right]$$ (13B)

Here, j is the index notation to denote the *j*th edge joint (crease connection) between *j*th and (*j*+1)th WB units. $U^{j}$ denotes the elastic potential energy of the *j*th unit [refer to Eq. (4)]. Now, the extra potential energy, resulted due to unit assembling, can be quantified mathematically by the term $U^{j*}$. Following a similar analogy concerning the above expression for 1D arrays, the corresponding expression for 2D metasurface will include a multiplication factor on the second term (associated with $U^{j*}$), since in a 2D assembly of arrays, each unit is typically surrounded by more than one unit. Similar to the normal creases (mountain and valley) of each unit, the interunit creases can also be analyzed by treating these as torsional springs with an equivalent spring stiffness $k^{j*}$. Here, the $\psi^{j}$ and $\psi^{j+1}$ denote the dihedral angles of the *j*th and (*j*+1)th WB, respectively, at their *j*th crease joint. The generation of $k^{j*}$ of the interunit torsional spring can be associated with the possible dihedral angle mismatch, resulted at the interunit connection of the array. The effective magnitude of $k^{j*}$ is related to many factors such as the assembled origami fabrication schemes and the material used. In the ideal scenario, a perfectly ideal rigid interunit crease connection can be described mathematically as $k^{j*}\to \infty$ [103].

Now, coming to the aforementioned dihedral angle mismatch, the compatibility condition states that during an entire rigid-folding process, the angles associated to the interunit crease should be identical, i.e., $\psi^{j}=\psi^{j+1}$, as all facets in the assembly are considered as fully rigid, and all creases act perfectly as hinges [46,104]. This theoretically makes $U^{j*}=0$, at each crease joint. As the inverting (reaction) force (F) can be related to potential energy as $F=-\nabla U_{s}$, the critical inverting force of the multiunit system during local and sequential stability state alteration of the WB units can still be calculated based on the total energy of the assembly during the motion stages. Note that the total energy is dependent on the crease angle deformation of all the creases forming the entire metasurface. However, considering that the energy contribution from the surrounding units are negligible, we only consider the energy associated with a single WB unit for calculating the inversion force without compromising working accuracy (confirmed further by experimental investigation later in the paper).

### Conceptual assembly for metasurface

*Origami architecture with locally controllable bistability.* A series of geometric models is analyzed to investigate how multiple WB units might be connected to form an array. The primary objectives of modeling are to investigate the interactions between adjacent WB units in such an array, to study the impact of changing the bistable state of a WB on the global curvature of the array, and to analyze the range of possible global curvatures the array can take on given the various input parameters (fold angle) and the state of local stability corresponding to each of the WB units. The procedure is outlined here for both WB6 and WB8 units, with a greater emphasis on WB8 due to its functional advantages as discussed later in the paper. Modeling is split into 3 key stages: the modeling of a single WB module, the modeling of a 1D array, and the modeling of a 2D array (refer to Fig. 1). Once the models are constructed, a curvature analysis is performed for the 1D and 2D array models (referred to here as metasurfaces).

The details of computer-aided design (CAD) models for WB units and their assembly are provided in Section S4. The method used to connect multiple WB8 units in a 1D array is to connect adjacent WB units at the edges and to introduce a rigid connection between the facets of adjacent WB units such that the facets remain parallel. In case of a 1D array of the WB6 units, the long edge of the larger facet is connected to the same edge on the adjacent WB. A rigid connection is again introduced between these facets such that they remained parallel. This eventually satisfies the required kinematic compatibility condition between neighboring WB units [46]. The basis for attaching multiple WB units requires that each individual WB remains as a quasi-rigid structure in its stable state. This means that all possible deformation modes of a single WB has to be represented by separate configurations of the assembly. This methodology allows the WB assembly to assume multiple different shapes, while at any time the assembly itself remains fixed in that state. This makes connection between adjacent WBs easy for a geometric model to calculate, as each WB shape is predefined due to bistability. For the WB8 unit, the deformation modes that are present in the 1D array occur due to the difference in the rest angle between the 2 bistable states. For implementation into a 1D array, there are 8 relevant deformation modes to consider (refer to Fig. S7B). The deformation modes in Fig. S7B, a to d, represent WB8-$M_{1}$ and the deformation modes in Fig. S7B, e to h, represent WB8-$M_{2}$. The concept of an interpolation angle $\psi_{\text{intp}}^{i}$, has been introduced here. Facets that fall on the boundary where a WB in $M_{1}$ is connected to a WB in $M_{2}$ will influence both geometries, which will result in $\psi_{v}$ falling somewhere between the rest angle for $M_{1}$ and $M_{2}$. The interpolation angle was chosen as the midpoint of the 2 rest angles for the remainder of this study based on experimental observation. However, if more accurate curvature analysis is required, further modeling and analysis would need to be carried out to determine the actual interpolation angle that could then be incorporated. For the WB6 WB, the only 2 deformation modes that can be present in the 1D array are the $M_{1}$ and $M_{2}$ configurations with $\psi_{v}$ set to $\psi_{v}^{M_{1}}$ and $\psi_{v}^{M_{2}}$, respectively.

The overall curvature of the assembly is obtained based on the stability states of individual constituting WB units that can be controlled independently. The connection method and the locally controllable switching between the deformation modes, discussed so far, is applied to a 1 × 16 WB8 unit array. This method allows the WB units to invert selectively and fully defines the curvature of the entire array. Two key factors influence the global curvature of the array: the inversion state of each WB ($M_{1}$ and $M_{2}$) and the rest angle ($\psi_{v}$) in each state. By independently varying these variables, the effect of each WB unit on the global curvature of the array is observed. Initially, the rest angles from the numerical formulation (refer to the “Analytical modeling of elementary origami kinematics” section), based on the geometric dimensions of CAD models, are used ($\psi_{v}^{M_{1}}=120\;{}^{\circ}$, $\psi_{v}^{M_{2}}=132\;{}^{\circ}$). Figure S7C shows a range of representative global curvatures achieved by using different combinations of inversion states with these rest angles. The model demonstrates that through different inversion patterns ($\begin{array}{c} A=\cup_{\left(i,j,\ldots ,k\right)}\left\{M_{i}^{1},M_{j}^{2},\;\ldots ,M_{k}^{N}\right\},\text{where}\;i,\;j\;,\ldots ,k\in \left\{\;,1,2\;\right\} \end{array}$), the array can achieve a range of different geometries. Because of the rest angles used, the WB in the $M_{1}$ state can result in an abrupt change in curvature. The different geometries ($2^{N}$ number of possible geometries, where N is the total number of WB units) that the structure can take is limited in order to prevent self-intersection. In reality, it is possible to achieve a wider range of rest angles by changing the stiffness of the creases and the weight of the facets and by prestressing the structure in either stable state. Figure S7D exemplifies another case of how, by altering the rest angles ($\psi_{v}^{M_{1}}=160\;{}^{\circ}$, $\psi_{v}^{M_{2}}=110\;{}^{\circ}$), more subtle curvatures in the structure are achieved. In a similar way, Fig. S7E presents the possible arrays based on WB6 units with nonzero $\psi_{v}^{M_{1}}$ and $\psi_{v}^{M_{2}}$. An assembly model is produced for the WB8 2D array as well, using the same connection method as proposed for the 1D array model. Figure S7F shows this WB8 array model with the key inversion combinations demonstrated. A WB6 model is not constructed for 2D arrays, as attaching the WB units along the edge of the shorter facet prevents the adjacent units from inverting.

In the above discussion, we have presented a few cases demonstrating the possibilities of achieving 1D and 2D curvatures (unidirectional and bidirectional) along with targeted discontinuous shapes. Notably, the scope of such curvature attainment increases significantly as the number of constituting WB units increases. The shape of the 1D or 2D domain, representing an effective surface, can be actively modulated to possess multiple globally stable shapes, by altering the stability states of individual WB units locally. In addition, it is possible to achieve a secondary-level assembly of multiple 1D WB arrays to form grids with single and multiple joints creating a vast domain of possible stable states (refer to Figs. 1E, e and f, and 2I and Fig. S7H, f). Thus, we essentially propose 2 different schemes of 2D active metasurface: (a) Primary metasurface assembly: 2D surface curvatures are obtained based on a continuous array of WB units that can be locally controlled to possess different individual stability states, (b) Secondary metasurface assembly: 2D curvatures can also be obtained by a secondary grid-like assembly of 2 or multiple 1D WB assemblies. Each of the WB units can be independently controlled to possess different local stability states. It is possible to attach 2 1D arrays in a single joint using WB8 (Fig. S7H, f), while a greater number of 1D arrays can be connected in WB units with more symmetric creases.

*Physical realization of the metasurface models.* Physical prototypes of the metasurfaces are investigated first using origami-grade paper models [101], followed by structural polymers. In the experimental quantification using paper models, the variables are taken as the thicknesses of the paper (120, 160, and 300 grams per square meter [GSM]), the number of creases (WB6 and WB8), the number of WB units in the surface to be investigated, and the method used to attach the individual WB units into arrays. Various thicknesses of single WB units are combined into several arrays by using cellophane tape. The other method used is folding a single sheet of paper into an array of constituent WB units. As in the initial modeling and theoretical analysis, a force is applied to the center vertex of one WB unit locally for the inversion to occur; a similar approach is also followed in the experimental investigation. Depending on the inversion state of the WB units locally, a global curvature would appear in the metasurface.

For the WB8 paper model, the mountain creases remain as mountain creases even after a force is applied to invert the WB unit to the other stable state, and the valley creases remain as valley creases as well. The only change in the structure during the inversion is that the angles ($\psi_{m}$ and $\psi_{v}$) between the creases and the origin would change. The total increment or decrement of the rest angles depends on the internal energy stored in the paper. Therefore, the greater the internal energy, the greater the inversion and the higher the angle of inversion. The WB6 structure also demonstrates a bistable behavior; however, applying a force at the tip does not cause inversion between visually similar-type states like WB8. Such varying phenomena can be understood based on a shift in the bifurcation path driven by the minimization of stored energy (refer to the discussion on the “Origami unit-level performance analysis” paragraph). The single unit model samples with different paper densities are shown in Fig. S7G, wherein a difference in the rest angles can be noted. Further, there is a significant difference in their inversion force due to the difference in energy barrier. The configurations with multiple WB units are shown in Fig. S7H, a to e, with single and double curvature. Interestingly, 2 1D assemblies can be connected in mutually perpendicular configurations with a common WB unit at the joint, leading to a gripper structure (Fig. S7H, f). Such WB-based grippers are able to capture objects of irregular shapes due to the locally controllable (conformal to the irregular object shape) bistability in the arms and achieve higher gripping force due to the bistable joint.

In the context of WB assemblies, one key difference of WB6 and WB8 units under consideration is in terms of the 1D and 2D metasurface construction. Because of the inconvenience in multidirectional control of WB6 units, an array of WB6 units cannot change its global shape in more than one direction due to the lower number of valley creases in a single unit. It can only bend in one direction when inverting (refer to Fig. 1C). Thus, WB6 under consideration is normally deemed suitable for 1D assemblies, whereas WB8 is suitable for both 1D and 2D assemblies. For this reason, we have focused more on WB8 assemblies in the current investigation.

After performing the computational analysis and paper-based modeling, a more in-depth investigation is carried out using engineering-grade materials, especially for accounting the rigidity of the facets. For that, polylactic acid (PLA) is chosen as it allows for quick and repetitive prototyping [105]. Note that PLA is not good as a crease material; however, it can be ideal for the facet design. The experimental investigation is performed on the level of both single unit and 1D array. For manufacturing of all the PLA facets, fused deposition modeling (FDM) is used through a Creality CR10-Smart machine with a 300 × 300 mm bed size. Further, Mylar-based origami models and their testing, to demonstrate polymer origami configurations without additive manufacturing, are accomplished to evaluate the effectiveness in applications like origami-inspired robotic gripper, with a proof of concept analyzed subsequently. For recording the force and displacement curves corresponding to bistability of single WB units and their assembly, an INSTRON 5569 electromechanical test machine (Instron Corp., MA, USA) is used [106] with 500 and 100 N load cells.

The response of a single unit is important for the comparison of theoretical data and finding the required inversion force. To accommodate this, the first step that needed investigation is the way to attach the facets of a single unit. A range of different crease materials are chosen and tested. The initially selected tapes (as crease material) are masking tape, duct tape, kinesiology tape, and high-temperature polyester tape. To understand the different properties of the tapes (with a constant width of 25 mm), initial tensile testing is completed, which involved cutting sections of the tapes and then applying an increased tensile force until the tape fails (refer to Table S3). From the recorded data, the Young’s modulus could be calculated, along with understanding how the tapes would react under a certain force. This information is utilized in the theoretical analysis. After this analysis, the high-temperature polyester tape was disregarded as its adhesive property was not as strong as initially thought. Besides tapes, it is demonstrated that paper-like material (300 GSM) can also be used as another crease component. However, the integration of the paper-like material within the PLA facet is done through 3D printing-based special facet designing techniques. The first technique involves creating a notch (C section) within the edge of the PLA facet, allowing the material (paper) to be inserted within and fixed with glue. The thickness of these facets is increased to 2.0 mm to allow the facets to be printed. The second technique involves using a Stop–Start (SS) printing technique. This entails designing and editing the center layer of the model to accommodate the crease material. The crease material can then be inserted within the samples by stopping the machine at the correct point and then continuing the print. The layers that accommodate the crease material have volumes continuing through it, stopping the material from slipping out of the facet. Both the fabrication techniques are depicted with the help of arbitrary triangular facets in Fig. S4 and shown in the context of the present WB8 unit in Movie S5. Such techniques allow for more flexibility when selecting the crease material. The testing results of single units and assembled metasurface models based on chosen crease materials are discussed in the “Origami metasurface level active programmability” paragraph.

### Numerical and experimental demonstration of active origami metasurfaces

**Fig. 3.**
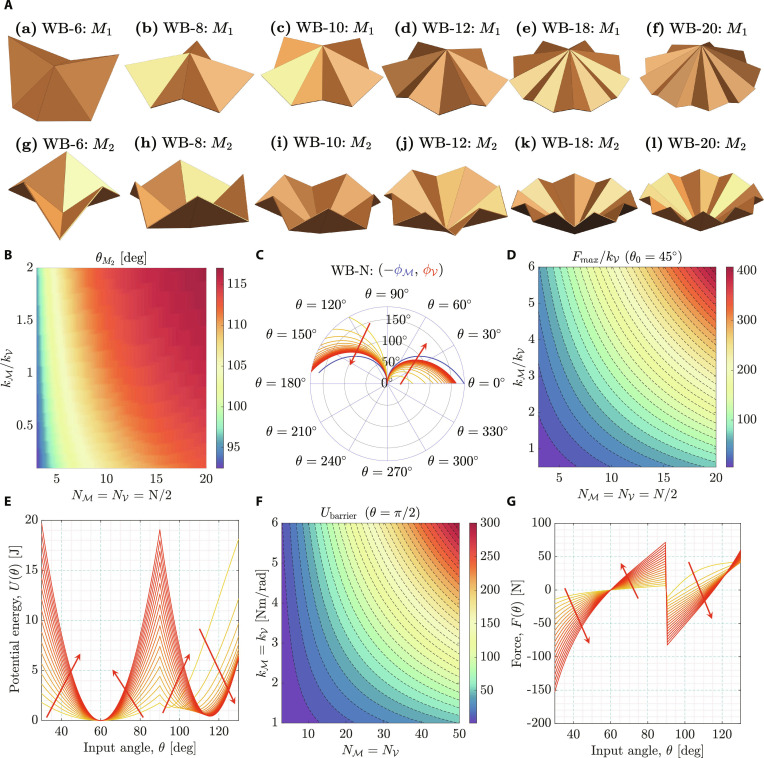
Stability insights of generic symmetric N-fold waterbomb units. (A) The stable configuration sets ($M_{1}$ and $M_{2}$) of N-fold waterbomb with N=6to20. (B) Contour plot of the stable angle corresponding to $M_{2}$ as a function of crease stiffness ratio ($k_{M}/k_{V}$) and number of similar creases ($N_{M}$ or $N_{V}$). Note that, here, the initial rest (stable) angle is set at $\theta_{M_{1}}=60\;{}^{\circ}$ for all *N*-fold units. (C) Variation of mountain (blue-colored) and valley (red-yellow graded lines) fold angles as a function of given angle θ for different N-fold waterbombs. In the case of the mountain angle ($\phi_{M}$), it is independent of the number of creases in the waterbombs. (D) Contour plot of the maximum inverting force for $M_{1}$ to $M_{2}$ configuration transformation as a function of crease stiffness ratio ($k_{M}/k_{V}$) and number of similar creases ($N_{M}$ or $N_{V}$). Here, the initial rest (stable) angle is set at $\theta_{M_{1}}=45\;{}^{\circ}$ for all N-fold units. (E) Variation of the potential energy [$U\left(\theta \right)$] as a function of given angle θ for different N-fold waterbombs with the same initial rest angle of 45°. (F) Contour plot of potential energy barrier (height of energy wall) between bistable configurations ($M_{1}$ and $M_{2}$) as a function of crease stiffness ratio ($k_{M}/k_{V}$) and number of similar creases ($N_{M}$ or $N_{V}$) with an initial rest angle of 60° (G) Variation of required force, $F\left(\theta \right)$ as a function of given angle θ for different N-fold waterbombs with the same initial rest angle of 60°. Note that in all the above figures, the red arrow (→) denotes an increase of waterbombs’ crease number.

*Origami unit-level performance analysis.* Here, we first discuss the theoretical analysis-based results (refer to the “Analytical modeling of elementary origami kinematics” section) at the unit WB level, taking a general *N*-fold WB as the starting point. The WBs are taken as rotationally symmetric and bistable in nature. The tunability of the bistable behavior is explored here by exploiting its various primary geometric parameters, particularly the number of creases, crease stiffnesses, and initial rest angle. Figure 3A depicts WB units with number of total creases varying from 6 to 20. In each case, the dimension is kept the same (for instance, the side length, r, and the number of mountain and valley creases, i.e., $N_{M}=N_{V}$). The apparent stable configurations corresponding to $M_{1}$ as well as $M_{2}$ are shown for each unit here (refer to Fig. 3A, a to f and g to l). The stable configuration can be evaluated based on the analysis of its stored potential energy due to the folding of WB units. Here, the amount of folding and motion behavior are quantified based on one single parameter (dof), which is θ. Figure 3C shows the changes in the dihedral angles corresponding to the mountain and valley folds with the change of θ. It can be observed that with the increase of crease numbers, an increase in the valley folds is realized within 0°<θ<90°, whereas the same trend gets inverted in the range 90°<θ<180°. Formation of any dihedral angle is not feasible in the range of180°<θ<360° because of the facet intersection. Now, regarding the mountain angle, the independence with θ is observed throughout its entire range. All the WB units hold the same mountain fold angle, validating Eq. (3A). Another interesting point to note here is that all the WB units hold the same (zero) valley fold angle at θ=0°; however, WB units permit different maximum values of θ at which the valley fold angle becomes 180°. For instance, the 6-crease WB permits a maximum range of θ up to 120°, whereas the unit structure with 10 creases provides θ=144°, where its valley fold angle becomes 180°. The reason for the existence of such a maximum limit of θ can be attributed to the same facet intersection at this region of θ. Figure 3C also validates the fact that fold angles become zero at the flat planar configuration (i.e., θ=90°) of the WBs.

According to Eq. (2), Fig. 3E plots the stored potential energy of different configurations from θ=0° to θ=180° of the WBs with crease number ranging from 6 to 40. Note that, here, as the initial rest angle is taken at θ=60° for all the massless WBs, the first stable state is found to be at 60°. However, there will be a shift of initial rest angle position and the first stable position if masses are taken into consideration, which we will discuss in a later section. With the increase of crease numbers, 3 important observations are noticed in Fig. 3E. Firstly, a consistent increase of potential energy with the number of creases is found throughout the $M_{1}$ region (0°<θ<90°); however, in the $M_{2}$ region (90°<θ<180°), especially after the second minima, this trend gets inverted. For instance, after θ=115.5°, all the WB units show a decrease in potential energy with increasing crease numbers. Secondly, the first stable configuration for all the WB is found exactly at its initial assumed rest angle ($\theta_{M_{1}}=\theta_{0}=60\;{}^{\circ}$); however, in the case of second stable configuration of WBs, $\theta_{M_{2}}$ is found to be shifted toward 180°. The higher is the number of crease (N), the higher will be the value of $\theta_{M_{2}}$ and the lower will be the potential energy corresponding to this stable position. In other words, increased creases reduces the asymmetric nature of bistability of the WB. For instance, the WB8 holds its second stable configuration with a potential energy of 1.265 J at θ=99°, whereas the WB40 holds the same with a potential energy of 0.432 J at θ=115.5°. The variation characteristics of second stable angle $\theta_{M_{2}}$ is discussed in more detail in Fig. 3B. Thirdly, an increased number of creases offers increased height of the energy wall (barrier), required to overcome for transition from the first stable configuration to the second stable configuration. The energy wall can also be termed the potential energy barrier ($U_{\text{barrier}}$), which will be found at θ=90°. This height of energy wall and its characteristics are further explored in Fig. 3F by varying not only the number of creases ($N_{M}=N_{V}=N/2$) but also the crease stiffness value ($k_{M}=k_{V}$). The trend suggests that as the crease number and stiffness increases, $U_{\text{barrier}}$ increases synergistically. This can be attributed to the increased individual equivalent spring energy and the number of spring, storing this energy. Figure 3B highlights the shift of the second stable angle as a result of change in the crease stiffness ratios and the crease number of the WB. It is observed that at the higher crease number, there is a decreased dependency of $\theta_{M_{2}}$ with stiffness ratio. On the other hand, when the stiffness ratio is low (<0.5), the change in the $\theta_{M_{2}}$ with respect to crease number is more pronounced in the lower value range of N/2 and eventually its value get saturated in higher value range of N/2. As the stiffness ratio increases, such trend is maintained, but the width of saturation zone increases.

Based on the potential energy characteristics discussed so far, the nature of forces required to invert the bistable shapes of WB can be drawn. Figure 3G shows the force–displacement profiles for the WBs with crease number varying from 6 to 40. Note here that the force (point load) is applied at the vertex of each WB along the negative *Z*-axis and displacement is quantified using the parameter θ. However, in actual case, the displacement (of vertex) is rcosθ, and the valley crease length, r, is set at unity in the present case study. The force can also be seen from another perspective, which is that here the force, $F\left(\theta \right)$, is the required force to keep the WB in the same configuration θ. According to this, the force required to keep the WBs in their stable configurations is zero, i.e., $F\left(\theta_{M_{1}}\right)=F\left(\theta_{M_{2}}\right)=0$. As the first configurations for all order WB is set at $\theta =\theta_{M_{1}}=60\;{}^{\circ}$ (refer to Fig. 3E), the required force is found to zero at this exact location in Fig. 3G. The higher is the crease number, the higher force is required for the transition between the 2 stable states. At a certain critical point (θ=90°), a snap-through phenomenon will occur under the application of a maximum force, $F_{\max}$. Similar to Fig. 3E, in the $M_{2}$ region of Fig. 3G, WB6 will reach the second stable state quickly, and the higher crease number is, the higher force is required for it to reach the second state. For the maximum transition force, $F_{\max}$, Fig. 3D presents a critical investigation on the combined effect of stiffness ratio and crease number. To enhance the applicability of the analysis, the force is normalized as $F_{\max}/k_{V}$ and stiffness ratio is normalized as ($k_{M}/k_{V}$). Both parameters offer a direct combined proportionate relationship with $F_{\max}$. While we present a detailed discussion of the architecture of symmetric WB units in this section, for the present study, we choose an 8-crease symmetric WB (N=8) so that a decent amount of influence of the stiffness ratio (refer to Fig. 3B) and of the required force for the bistable transition can be maintained (refer to Fig. 3G).

**Fig. 4.**
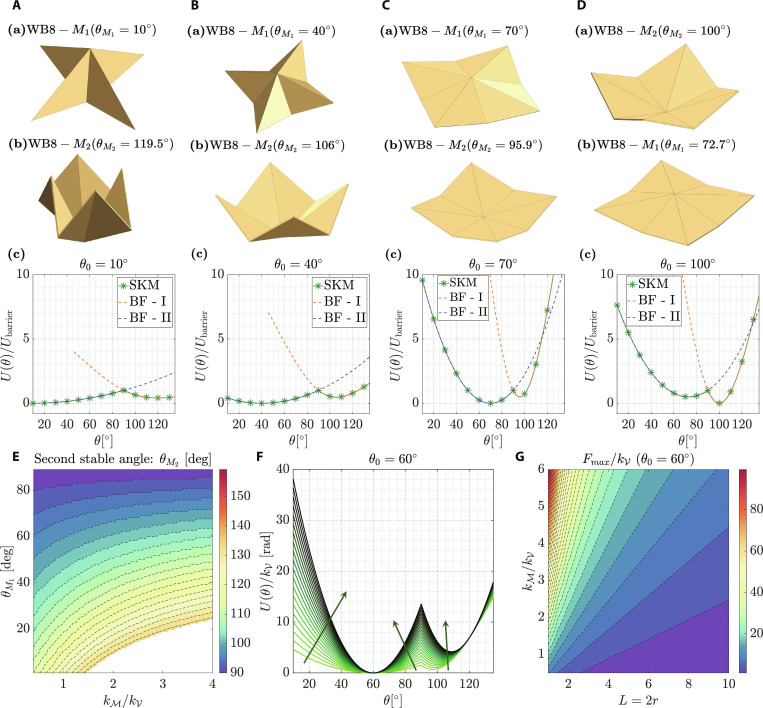
Stability states of symmetric 8-crease waterbomb units. (A to D) The bistability of WB8, predicted by the spherical kinematic method (SKM) [81] and bifurcation theory (BF) [42] is shown by plotting the potential energy ratio as a function of angle θ for the 4 different initial rest angles ($\theta_{0}$). The bistable configurations [i.e., (a) and (b)] of the waterbomb corresponding to $\theta_{M_{1}}$ and $\theta_{M_{2}}$ are shown for all 4 cases. The 4 rest angles assumed here are as follows: (A) $\theta_{0}=10\;{}^{\circ}$, (B) $\theta_{0}=40\;{}^{\circ}$, (C) $\theta_{0}=70\;{}^{\circ}$, and (D) $\theta_{0}=100\;{}^{\circ}$. The last rest angle highlights the case of configuration transformation from $M_{2}$ to $M_{1}$. (E) Contour plot of the stable angle corresponding to $M_{2}$ as a function of the first stable angle ($\theta_{M_{1}}$) and the crease stiffness ratio ($k_{M}/k_{V}$). Because of neglecting the effect of the mass factor of the waterbombs, the first stable angle can be calculated from the initial assumed rest angle, θ. Note that the empty region of the contour plot highlights the infeasible geometry due to facet intersections. (F) Variation of the potential energy [$U\left(\theta \right)$] as a function of given angle θ for different stiffness ratios of waterbombs ($k_{M}/k_{V}=1\cdots 6$) with the same initial rest angle of $60^{\circ }$. The arrow (→) highlights the increment direction of the stiffness ratio. (G) Combined influence of the crease stiffness ratio and the dimension (side length, L) of the waterbomb square on its maximum inverting force, $F_{\max}=F\left(\theta =\pi /2\right)$, required for the bistable state alteration.

Figure 4 presents an in-depth investigation considering 8-crease WB (WB8) to analyze the effects of influencing parameters. Figure 4A to D highlight 4 cases with 4 different initial rest angles ($\theta_{0}=10\;{}^{\circ},40\;{}^{\circ},70\;{}^{\circ},\text{and}\;100\;{}^{\circ}$), but with the same dimension (r=L/2=1). Note that L is the side length of the unfolded flat WB8 unit. The potential energy utilizing the spherical kinematic model [36] and the bifurcation theory [42] is plotted against its single dof (i.e., θ). It can be noted that out of 2 permissible bifurcation paths, the structure will tend to follow the one with less energy path and the bifurcated point is obtained exactly at θ=90° in all 4 cases. The resultant final path is predicted quite well by the spherical kinematic model. However, the bifurcation theory is, in general, more accurate in predicting the motion path based on its potential energy profile than the spherical model. For instance, in the last 2 cases with $\theta_{0}=70\;{}^{\circ}$ and $\theta_{0}=100\;{}^{\circ}$ (refer to Fig. 4C and D), the structure will tend to retrace and follow the bifurcation path II after θ=116.4° and θ=129°, respectively. However, the spherical kinematic model fails to predict these phenomena here. Figure 4A and B, a and b, show the 2 stable configurations for all the 4 cases and their corresponding stable angle ($\theta_{M_{1}}$ and $\theta_{M_{2}}$), calculated from the potential energy profiles in Fig. 4A and B, c. Figure 4D, a to c, highlights one case where the initial stable configuration ($\theta_{M_{2}}=\theta_{0}=100\;{}^{\circ}$) is taken in the $M_{2}$ region and shape transition happens from $M_{2}$ to $M_{1}$, whereas in rest three, a typical left to right direction ($M_{1}$ to $M_{2}$) along the potential energy profile is presented. Analysis on the third scenario ($\theta_{0}=60\;{}^{\circ}$) is further detailed in Fig. 4F and G. Figure 4E presents a contour plot highlighting the interplay between the crease stiffness and the initial stable angle in determining the second stable angle. The crease stiffness is given here as a stiffness ratio to capture the simultaneous variation of valley and mountain stiffness in a 2D contour form. To show the numerical results, the valleys are considered to possess unit stiffness. The initial stable angles ($\theta_{M_{1}}$) near the flat configuration (θ≈90°) consistently lead to a second stable configuration that is also close to this flat configuration. As $\theta_{M_{1}}$ moves further away from the flat, unfolded configuration, $\theta_{M_{2}}$ similarly deviates more from it. On the other hand, a higher stiffness ratio results in a higher value of $\theta_{M_{2}}$. However, $\theta_{M_{2}}$ can only be maximized up to 135° due to its self-contact folding boundary condition [46], as shown by the empty region of the contour plot, beyond which the geometry becomes unfeasible to achieve. Figure 4F pinpoints a specific case of $\theta_{M}=60\;{}^{\circ}$ from this contour plot, highlighting the potential energy profile at all the points of this line. In other words, the variation of the potential energy stored in all the permissible configurations (0≤θ≤135°) of the WB with crease stiffness ratio ranging from 1 to 6 is plotted. It has been noted that the influence of stiffness ratio is more pronounced up to the second stable angle; afterwards, the relative influence has reduced substantially. The second stable configuration shifts toward the flat planar configuration as the stiffness ratio decreases, which also validates the observation obtained in Fig. 4E. In essence, a lower stiffness ratio results in weak bistability in the system. Finally, based on this potential energy profile, the peak inverting force is calculated and explored under the combined influence of the WB’s overall dimension (side length, L) and stiffness ratio ($k_{M}/k_{V}$) (refer to Fig. 4G). Such investigations in terms of stiffness ratio and dimensions can particularly help in the selection of materials compatible with the adopted actuation mechanism (magnetic actuation is discussed later in this paper).

Figure S8 presents a discussion on the rotationally asymmetric 6-crease WB (under consideration in this paper) and its bistability. Figure S8A to D show the bistable configurations, evaluated based on the comparatively more accurate bifurcation theory. As mentioned during the discussion on WBs without rotationally symmetric geometry, unlike WB8, the configuration of WB6 can be better described by the generalized coordinate $\theta_{f}$, instead of only θ. This is because θ becomes fixed at 90° in the $M_{2}$ state, while $\theta_{f}$ varies continuously and uniquely with each configuration during the folding process of the WB6 unit, regardless of whether it is in the $M_{1}$ or $M_{2}$ state. $M_{1}$ and $M_{2}$ states offer 2 nonidentical stable configurations where θ becomes 90° in the $M_{2}$ state for almost all the cases. The initial rest angles in all 4 cases and angles corresponding to their stable configurations are described by both the angles ($\theta_{f},\theta$). The strength of bistability decreases with the increase of the initial stable (rest) angle $\theta_{f}^{M_{1}}$. Figure S8D shows the scenario with the weakest stability among the 4 configurations. Figure S8E shows the relationship of 2 dihedral angles of the mountain ($\psi_{M}$) and valley ($\psi_{V}$), and the alternate position angle (θ) with the generalized coordinate angle $\theta_{f}$. Following a parabolic increase in the $M_{1}$ state, a plateau stage of θ=90° is achieved throughout the $M_{2}$ state of the WB6. A similar observation is found in the case of $\psi_{M}$ as well, where a plateau stage of 180° is achieved. It is also evident from the second stable configurations of the WB6 in Fig. S8A to D, b, where the fold angles in all 4 mountains become zero. Now, regarding the valleys’ dihedral angle, a continuous variation throughout the state (in $M_{1}$ and $M_{2}$ both) is obtained except at $\theta_{f}=90\;{}^{\circ}$, where a sharp jump in its value is noticed. Figure S8F and G highlight the potential energy and the inverting force profile for the WB with $\theta_{f}^{0}=60$ (refer to Fig. S8D), for different stiffness ratios and the side lengths. A similar trend is observed as Fig. 4F and G. It is important to note that the $U_{\text{barrier}}$ and $F_{\max}$ of WB6 are seemingly higher than those of WB8. However, it is not strictly true as these 2 parameters are plotted within the generalized coordinate, $\theta_{f}$, not in θ. If a true comparison is carried out for the WB in θ coordinate only, WB8 (with 8 creases) is expected to have a higher energy barrier between the stable states and require a higher inverting force compared to WB6 (with 6 creases). For instance, in the case of the initial rest angle θ=36.869 in both the WB, the $U_{\text{barrier}}$ in WB8 is found to be 69.89% higher than that in WB6. This is a result of the increased number of creases and the resultant geometry of the second state of the WB8 being more stable compared to the WB6.

So far, the effect of facet mass has not been accounted for in the presented numerical results. Here, we have shown the influence of the facet masses on the initial rest angles of the WB. In the preceding results, it has been observed that the first stable angle resides exactly at the same assumed rest angle; in other words, $\theta_{M_{1}}=\theta_{0}$. This equality sustains in the case of WB with negligible masses. However, there will be a shift of the first stable angle from the initial rest angle in the case of WB facets with nonzero masses. Note that, in the latter case, the assumption of rigid facets is still taken care of. Based on the analysis given in the “Remarks on rest fold angles ($\psi_{M}^{R},\psi_{V}^{R}$) for the WB” paragraph, Fig. S9 shows 2 case studies for the WB8 and WB6 each, where the 2 influential parameters (crease stiffness and facet masses) are varied simultaneously to see how it affects the rest angle. For the WB8 (refer to Fig. S9A and B) and WB6 (refer to Fig. S9C and D), the initial rest angles $\theta_{0}^{p}$ (which is, in general, a result of residual plastic deformation in sheet-based origami or the free-leg position of torsional spring in origami-based mechanical structure) are taken at 45°. All the cases shown in Fig. S9 illustrate that an increase in the facet’s weight ($m_{f}$) tends to stabilize the origami unit toward a rest configuration close to the flat planar state ($\theta =\theta_{0}^{\;f}=90\;{}^{\circ}$), whereas an increase in crease stiffness ($k_{M}$ or $k_{V}$) resists this tendency, wherein an interplay among the crease stiffness, facet mass, and dimension becomes evident. Figure S9E and F, on the other hand, show 4 different masses of magnetic setup that are implanted onto the vertex of a WB8 unit with 2 different sizes. It can be observed that as the mass of the setup is increased, the first rest configuration tends toward a flatter configuration, which corroborates our intuition as well. Further, with the increase of magnet masses, the possibility of getting rest configurations with lower crease stiffnesses has also reduced.

Before investigating the metasurface level behavior, we have carried out a few initial experimental testing at the unit cell level (refer to Fig. S10A), comparing different samples with each other to give a brief overview of the bistability along with the inversion force (primarily focusing on the inversion from $M_{1}$ to $M_{2}$). To allow the models to be tested in an INSTRON 5569 electromechanical test machine, a range of jig configurations and designs are examined to find the optimal design. In such experimental testing, jigs are required to constrain the model, stopping it from moving around or applying force onto the structures at a particular point. To keep the models in place but allow the inversion to occur, few sufficiently rigid jigs with a hollow center (Central Inversion Cavity) are chosen to be printed out of PLA that meet the strength requirement (refer to Fig. S11). To apply the force to the unit, 2 different sized jigs are finally used for the initial and final testing due to the attachment size difference between the 500 N and the 100 N load cell. The jig setup of the initial and final testing is shown in Fig. S10B. Because of the pointed shape of the WB central vertex and the initiation of snapping-based state inversion before reaching perfect flat position, a small diameter of the pressing rod does not affect our ability to apply an effective point load here. Note that, here, the term “initial testing” refers to the testing in which the force and displacement curves for all WB sample types (refer to Fig. S10A) are recorded using a 500 N load cell. Based on these results and the criticality of the inversion responses, a few selected types are later narrowed down and tested more precisely using a lower load cell, i.e., 100 N, which is referred to here as “final testing”. For instance, in the initial testing, the maximum force before inversion is identified for each sample, along with the displacement it occurs at (refer to Fig. S10D). From these results, a range of different critical aspects can be identified. Firstly, the double-layered masking tape requires a significantly greater inversion force when compared to the other samples. For instance, this force is 194.562% higher than the force in those with the single-layered masking tape. This shows that the stiffness of the crease material will significantly alter the force required to invert the sample. This concept is also shown by the difference between the 2 C-section samples, which had a different thickness of paper. The thicker and stiffer paper resulted in a greater inversion force. Secondly, from these results, the kinesiology tapes start inverting at a lower displacement than the rest of the samples. This is likely due to the stretchy property of the tape forcing the structure to invert, allowing it to return to its original prestressed state. This shows that, by prestressing the crease material in targeted mode, the state change of the structure will be more pronounced. From this analysis, the double-layered masking tape is chosen to be tested further. The double-thickness paper model is also chosen to be tested further but with 300 GSM paper. The SS samples additionally give the highest precision for inversion force, which is desired between samples when applied in an array. This is likely due to the samples having a constant crease thickness, as the sample is printed as a whole unit rather than manufactured as individual facets like all the other C-section samples and tape samples. For the final testing of the single units, 3 repeats are manufactured for the 2 different WB units; additionally, each repeat is tested 5 times. The different samples tested are shown in Fig. S5. As mentioned previously, for the final testing, the 100 N load cell now is utilized. This allows for more accurate recorded data and trends. The initial and final force–displacement tests, corresponding to Fig. S10B, are shown in Movie S1. The inversion force required for both stable states ($M_{1}$ and $M_{2}$) is investigated to be able to compare the difference. The results concerning the maximum force before inversion for each sample are shown in Fig. S10E and F for $M_{1}$ and, $M_{2}$ respectively. From these results, the SS WB units continued to show high repeatability between the samples and between the individual test of each sample. For the double-layered masking tape samples, a significantly greater force is still recorded when compared to the SS samples; however, the repeatability between samples is significantly lower. This is due to the samples not having an even crease thickness due to the movements of the facets during manufacturing. When comparing the inversion force required for both states, the samples show a similar trend for inversion from both states. For the double-layered masking tape samples, there is more overlap between the sample tests in $M_{2}$ but showing lower precision. The force to convert the samples from $M_{2}$ to $M_{1}$ requires significantly less force than the opposite state change. Note that fatigue is an issue that is seen within the testing of each individual sample. Before each individual test, the samples are prestressed and folded to make sure the effect of fatigue is minimized. For the final crease material, double-layered masking tape is chosen due to the higher inversion force required to change its state. Even though the repeatability between samples is not as high, due to the bistable nature of the WB units, high precision is not needed in the application of external stimuli for generating the critical inversion force. This is one of the major advantages of the current work in obtaining targeted shape modulation of the metasurfaces.

Finally, at this stage, validation of the analytical results in case of a single WB8 origami unit is performed against the finite element analysis (FEA) results and the experimental mean data from the sample (refer to Fig. S10C). The details regarding the FEA implementation are given in Section S5. Note that, for the experimental data, the one with the 3D printed PLA and SS variation is considered. This is done to get the most accurate comparison, as both the analytical and numerical results assume rigid facets and perfect torsional springs as the creases. The experimental data show a good correlation with the analytical and FEA data, although the exact value of crease stiffness could not be determined precisely as it is difficult to manufacture the SS units with exact crease widths. Despite this, the maximum force (~0.5 N) value found is very similar, indicating the robustness of the analytical framework discussed in the paper in predicting the inversion force.

**Fig. 5.**
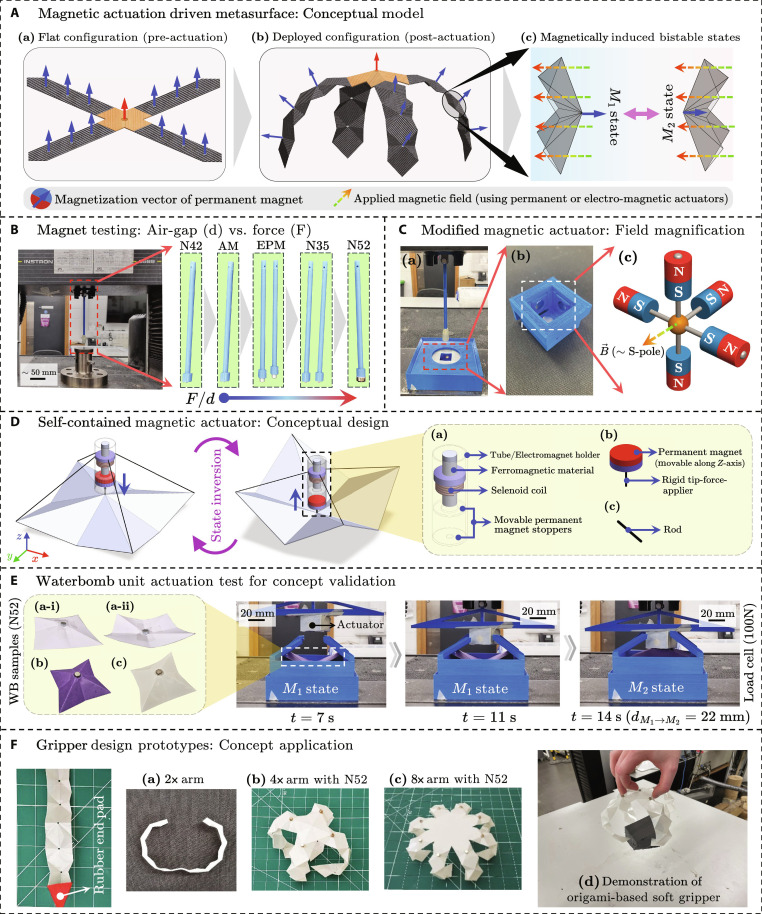
Active programmability of the multistable states in origami metasurfaces. (A) WB8 assemblies with vertex magnets actuated by an external magnetic field. (B) Magnet strength test setup (air gap vs. force). (C) Magnetic actuator: (a) strength test setup, (b) polarity-switching design, and (c) Bushman-type amplified magnet [108]. (D) Conceptual design for self-actuation via an internal magnetic source. (E) Snap-through testing of 100 mm × 100 mm waterbombs with embedded N52 magnets: (a) 120 GSM paper (i. $M_{1}$, ii. $M_{2}$), (b) 160 GSM paper, and (c) Mylar. (F) Mylar-based gripper prototypes (a to c) 2-, 4-, and 8-armed grippers and (d) soft robotic gripping application for an irregular object.

*Origami metasurface level active programmability.* Figures 1E and 2G to I demonstrate the active modulation capability of 3 separate kinds of origami metasurfaces through sequential and algorithmic alteration of local stability states of the WB units: (a) 1D arrays, (b) 2D arrays, and (c) grid-like secondary architectures of multiple 1D arrays. It becomes evident from the figures that 1D arrays can assume a range of curvatures and shapes depending on the local stability states of each WB unit. Likewise, different unidirectional, bidirectional, and other target shapes are achievable in the continuous 2D array of WB units and grid-like secondary architectures. In this context, the aspect of postmanufacturing active shape modulation in a single specimen should be noted. Interestingly, the secondary architecture with one joint leads to the design of origami grippers, as shown in Figs. 1D and 5A and F. A critical feature of such grippers is the ability to hold irregular objects due to the capability of controlling the local stability states in each of the arms (i.e., conformability to the shape), in addition to controlling the stability state of the joint that results in the gripping action. In this context, we would like to emphasize that the focus of the present paper is to propose a framework for modulating the geometry of origami-architected 1D or 2D surfaces, where metasurfaces, gripper, and robotic crawling are presented as potential applications. In other words, the gripper is presented here primarily as a proof-of-concept application of the programmable sequential state-alteration strategy developed in the present work, rather than as a dedicated study of force-optimized grasping. Based on the kinematic and energy descriptions, together with the further geometric and kinematic formulations, the gripping force is expected to depend on (a) the number of origami units in the gripper arm, which determines the overall compliance and achievable curvature for a given configuration of inverted states, and (b) the geometric parameters and crease stiffness of each unit, which govern the unit-level resisting moment and stored elastic energy during switching, and therefore influence the contact force transmitted to the grasped object. To clarify this dependence while maintaining the manuscript’s focus on programmable deployment, we have provided a general energy explanation to rationalize the force trends in Section S3.

In the preceding sections, we have discussed the computational and experimental investigation of the bistable nature of WB origami units, which is the basic building block for developing 1D and 2D metasurfaces. Here, we first focus on the experimental demonstration of multiunit WB architectures to assess the effect of surrounding units. Therefore, the test is carried out on a 1 × 3 array considering 2 cases: in the first case, the force is applied to a single unit of this array; in the second case, the force is applied evenly to all the units of the array simultaneously and thereafter the force for one single unit is calculated by dividing the total force by the number of units. The model test for the second case is shown in Fig. S10H and Movie S2. Note that, for the 1D array testing, a new base and top jig are manufactured as the base jig needed to be able to accommodate 3 units. During testing, the force–displacement responses are recorded. The models in the array are kept within the same state (here, only $M_{1}$ is investigated) before the application of the force. Figure S10G shows that there are no significant effects on the inversion force values when units are attached in an array compared to the testing of a stand-alone single WB unit. Note that in Fig. S10G, by the term “1D Multi”, we refer to the aforementioned second case, whereas the term “1D Single” refers to the first case. Such observation allows the single unit force to be taken as the base required force when magnetic actuation is applied, as discussed in the following section.

Another 1 × 14 WB array is manufactured to understand the behavior of the array depending on the local stability state of each unit. Figure S10I shows some of the possible geometries (that can be actively modulated in a single specimen) depending on the stability state of each unit. Figure S10I, a and b, show the array when all the units are in the same state ($M_{1}$ or $M_{2}$). When all the units are in $M_{1}$, the angle of curvature is greater than when all the units are in $M_{2}$—this is due to the rest angle of respective states. By having a combination of states, the models can have significantly different geometries (refer to Fig. S10I, c and d). When one unit changes its state, there is a noticeable geometry alteration to the neighboring unit, considering the new angle between the connecting facets.

For the 2D modeling of metasurfaces, 3 different attachment methods are experimentally investigated (refer to Fig. S6). In the first attachment method, the masking tape is used to attach the center of each facet of the 1D arrays. In the second attachment method, masking tape is used to attach the outer corner vertices of the facets of each unit in the 1D array. In the third attachment method, the earlier 2 methods are merged simultaneously, where the masking tapes are used to attach the center of each facet in both directions. Each method is analyzed by inverting different units to see the behavior and the limitations of the attachments. For instance, in the third method, the complete inversion of a singular unit is stopped within the array, with only inversions of more than one unit being possible. If more units are attached to the array, the units that are within the center of the array would not be able to invert. For the first method, as the facets are attached in the middle, this restricts the separation movement of them when the valley creases would decrease in angle. This means the rest angle would not be able to change as before. The second method also has this restricting movement, although this is not significant. From this initial analysis, it is shown that the attachment method must not restrict the movement and motion of the units. From the previous single-unit crease material selection, kinesiology tape provided the flexibility and strong adhesive property, which are required for the attachment of the 2D array. Kinesiology tape is then applied following the second method onto a 4 × 4 array. The motion behaviors depending on the state of individual units are shown in Fig. S10J. The configuration with all units in the $M_{1}$ stable state shows more global curvature compared to the case when all units are in the $M_{2}$ stability state, primarily due to their respective rest angles.

So far, in this paper, we have (a) investigated the bistable states and required inversion force for WB units (WB8 and WB6) and (b) demonstrated the ability of achieving a range of different curvatures in 1D and 2D arrays of WB units through algorithmically controlling the stability states of the constituting WB units locally. In the following subsection, we will discuss a contactless approach of controlling the local stability states through magneto-active actuation that can be algorithmically programmed from a remote location to achieve a range of target shapes (on-demand) using a single specimen.

*Far-field magneto-active control of metasurface shape and curvature.* As previously noted, the central vertex of a WB unit only needs to move along the *Z*-axis when inverting from configuration $M_{1}$ to $M_{2}$, or vice versa. This key insight allows for the implementation of far-field magnetic actuation [95,107] that follows the WB’s central vertex, provided that the movement of the WB is properly constrained. Specifically, all sides of the WB must be limited to restricted movement along the *X*-axis and *Y*-axis during actuation, in line with the conventions used in the theoretical framework. Such assumptions hold good in the current framework of tessellated WBs with reasonable extent for applying the analytical results concerning critical inversion force. It may be noted in this context that the proposed piece-wise distributed bistable actuation mechanism allows a significantly higher flexibility in terms of the accuracy demand of critical inversion force compared to conventional monostable structures while targeting a specified shape. As long as the applied actuation force is higher than the critical inversion force and it does not affect the structural integrity, the proposed metasurface would function as per target objectives. Given that permanent magnets have 2 poles, a permanent magnet is attached to the central vertex of a WB model to observe its behavior in response to another magnet (or equivalent external magnetic field) held close by. By controlling the polarity of the external magnets, the embedded magnet can experience force (either an attractive or repulsive), which forms the basis of the magnetic actuation.

Seven different types of permanent magnets, illustrated in Fig. 5B, are studied and analyzed, as detailed in Table S4, before selecting the final magnet for the actuator. These magnets chosen are then tested with the setup shown in Fig. 5B. One magnet is secured to the base of the INSTRON 5569 machine, while the other magnet is secured to the moving part on top. The distance is then varied to obtain the relationship between air gap distance against force (refer to Table S5). Note that some of the results are not available because the forces are too small for the load cell to record. Assemtech Magnet, N42 Magnet, and N35 Magnet(a) are removed completely from the results as none of the data could be recorded for the range of air gap distance. The results show that the N52 Magnet gives the best results because it provides sufficient force for a wide range of air gap distance. Therefore, it has been decided to proceed with the N52 Magnet. In this context, it may be noted that we have used a permanent magnet for its simplicity in demonstrating the concept. However, more sophisticated and powerful external magnetic sources based on electrical coils (electromagnet) can be adopted depending on the inversion force magnitude—following similar principles as discussed here.

The active magnetic control is essential for dynamically managing the magnetic gripper or origami structure. For that, initially, a normal electromagnet (electromagnet consisting of a coil of wire wrapped around a ferromagnetic core) is tested to check the possibility of producing similar magnetic forces within certain air gap distances found in the case of the embedded N52 magnet (refer to Table S5). Note that, here, the electric current is taken as an external variable factor to control the strength of the produced magnetic forces. It is found that within the available range of electric current in the testing laboratory, the produced force is not enough to invert the stable states of the WB. Such a finding can be attributed to many factors, such as the typical reduction in magnetic field density that occurs with distance in standard electromagnets, decrement in the current due to increment of the coil resistance with heat, etc. While we have not proceeded with using electromagnets for the current tests (since our objective here is to demonstrate the possibility of magneto-active stability state inversion only; the method of achieving the magnetic field is not the current focus), this remains an attractive way depending on the available range of current and coil parameters. To overcome this, a special setup utilizing the concept of amplified magnetic beam [108] is introduced (refer to Fig. 5C, c). According to Bushman [108], arranging 5 permanent magnets with the same poles pointed toward the center can significantly amplify a magnetic beam and can counter the loss of magnetic field density in normal electromagnets. This solution is ideal as a focused linear magnetic field beam aligns best with the magnetic actuation requirement of displacing the WB vertex in the *Z*-axis direction. Furthermore, by using this method, we have eliminated the need to utilize bigger and stronger permanent magnets, where the magnetic strength would have remained underutilized and undesirably interact with the surrounding unit cells. Based on the concept of amplified magnetic beams, a more precise control on the sequential stability state alteration can be achieved. Manipulating weaker magnets to fully utilize the pull force is better than only using a small percentage of an excessively strong magnet, which would pose safety hazards such as pinching of skin as well as making it impossible to use it near electronics and machines.

As shown in Fig. 5C, b, a setup is designed to hold the N52 magnets in such a way that resembled the above method [108]. In addition, this design has the flexibility of switching permanent magnets’ poles to generate a north or south pole magnetic beam, which would be used to generate an attraction or repulsion force accordingly. A test is then conducted to find out the attraction and repulsion force generated by the magnetic actuator. The magnetic actuator is secured to the bottom of the INSTRON 5569 machine, while another N52 magnet is secured to the top of the machine and attached to a 100 N load cell. The results are then recorded in Table S6. The results prove that the developed magnetic actuator design shows a significant increase in magnetic force (either an attractive or repulsive) compared to the force produced by the single N52 magnet. With the force produced being sufficient to invert the WB model embedded with an N52 vertex magnet, we thereafter proceed to the integration of magnet to the origami and assembly stage.

After designing and gathering the necessary force data, a permanent magnet is incorporated into the WB models. The magnet is first attempted to be secured at the vertex of the WB models using cellophane tape, although this approach leads to 3 issues. First, the tape stiffens the creases of the WB, increasing the force required for inversion. Second, the tape restricts the movement of the WB, resulting in a slight alteration of its bistability (refer to Fig. 5E, a). Third, the tape is not strong enough to hold the magnet securely, leading to uncontrollable displacement and rotation of the magnet under magnetic forces. Figure 5E, b, shows the implementation of the N52 magnet, secured using superglue in a 100 mm × 100 mm WB vertex. Superglue is an ideal choice because of its sufficient strength to hold the magnet in place without significantly affecting the WBs creases or its bistability. Note that a hole is cut in the center of the paper to accommodate the magnet. Such a hole has minimal effect on the WB bistability. Afterwards, a support structure (as shown in Fig. 5E) is designed to restrain the WB movement so that the vertex can only move along the *Z*-axis while the sides can only move in-plane. A test is performed using this structure to observe if the magnetic actuation concept is working. The test is carried out by securing the magnetic actuator on the top of INSTRON 5569 and the support structure is secured to the base, with a WB placed in the support structure. The top part of the machine is then slowly lowered until the WB is inverted, and then the air gap distance is recorded. This test is presented in Movie S3. The test reveals that the 160 GSM WB manages to invert from $M_{1}$ to $M_{2}$ when subjected to a repulsive force at 22 mm and from $M_{2}$ to $M_{1}$ when subjected to an attraction force at a 46.3-mm air gap distance. Following this test, an N52 magnet is also embedded in the Mylar WB with the same setup as the 160 GSM paper WB, and the test is repeated, as shown in Fig. 5E, c.

In the preceding paragraphs, we have demonstrated experimentally that the stability state of a WB unit can be altered based on far-field magnetic actuation. Such a concept can subsequently be implemented in assembled arrays of multiple WB units for achieving on-demand target curvatures through algorithmically altering the local stability states in metasurfaces (refer to Figs. 1E and 2G to I). To this end, different prototypes of the robotic gripper are developed based on the concept of secondary assembly to show a potential application of the present work. Such designs include the 2-, 4-, and 8-armed gripper. It may be noted in this context that such soft origami robotics are inherently suited for the interaction with delicate materials, and their conforming characteristics are generally able to exert compliant forces, unlikely to damage the object.

The present armed grippers are essentially envisioned as 1D array, or an assembly of such arrays, with the number of WB units adaptable to match the gripping force and object dimension requirements. However, the units (at the end of gripper arms) are not ideal as tactile gripping surfaces. Therefore, to achieve effective gripping, an extended triangular-cut origami fold end is utilized, which can be used as a spike grip directly (see Movie S4), or can have a small rubber pad attached to provide a larger tactile surface (Fig. 5F), ultimately forming a very rigid lightweight end section. As shown in Fig. 5F, a, a 2-armed gripper using Mylar is created consisting of 10 WB units and a rubber pad can be attached at each end in the aforementioned manner. Only having 2 arms allows stabilization of the load in only one axis, and the soft nature of the structure makes it unable to function properly. To achieve better stability, higher numbered arms are designed. For instance, 4- and 8-armed grippers are 2 and 4 1D arrays connected at a central point. For these designs, smaller-sized N52 magnets at the central vertices of the innermost units on the arm are integrated (refer to Fig. 5F, b and c). The first unit in each arm is the best location for the actuation of individual arms. Various actuation points can be used, wherein the central unit can move all the arms simultaneously in the 4-arm configuration along with additional local stability control in each of the arms. The 8-armed gripper demonstrates significantly higher lifting capability, but the design does not have central unit stability control. Note here that the Mylar prototypes are created using a specific origami/Kirigami cutting machine named Silhouette Cameo 4. Based on manufacturing ability, the proposed concepts can be extended to different sizes of the grippers as per the requirement. As shown in the prior parametric studies (refer to Fig. 4G and Fig. S8G), decreasing the size of the units leads to a higher inverting force because of the reduced moment arm size. In other words, scaling down the gripper unit size requires either the scaling up of the magnetic force needed to actuate the unit inversion or the reduction of the distance between actuating magnet and the units. The gripper prototypes shown in Fig. 5E can be actuated preferably at very close ranges, due to the fact that the units are created with smaller side lengths (around 33) in the Silhouette Cameo 4 machine.

In the current demonstration (refer to Fig. 5F, d, and Movie S4), the gripping force is strong enough for the 4-armed gripper to grip and lift reasonable weight. Analytical energy-based insights into the gripping force quantification are provided in Section S3. Interestingly, smaller grippers can lift several times their own weight more easily due to the increased inverting force. The present active far-field actuation strategy provides a scope of dealing with much higher weight objects by on-demand increasing the inversion force through the application of additional magnetic field during the lifting operation. Figure 1D presents conceptual schematics of a few gripping modes for the soft grippers, which can be achieved by rationally placing magnetic actuation centers in present WB based gripper designs. For instance, the transition between quadruple contact mode and the neutral no-contact mode in the 4-armed gripper can be achieved just by the inversion of the central unit, similar to Movie S4. On the other hand, multiple-unit inversion in the same gripper can enable the distributed mode, leveraging multiple contact points across a soft surface, distributing the force, and allowing for stable grasping of objects with complex shapes. Moreover, the precision pinch mode is also possible, which utilizes a small, focused contact area, typically at the fingertips, to achieve precise grasping and manipulation.

In the current context, we would like to highlight that the focus of the present paper is to propose a framework for modulating the geometry of the origami-architected 1D and 2D surfaces, where the gripper is shown as a potential application. In the experiment demonstrated in Fig. 5E, we have illustrated that bistable state inversion can be achieved with the applied magnetic force with certain threshold nonzero values. Although we used permanent magnets in this proof-of-concept test, the key point is the existence and magnitude of the required (threshold) magnetic force, rather than the specific choice of magnet. For instance, it is mentioned that N52-based magnet strength at a distance around 22 to 46.3 mm from the vertex is capable of state inversion. This result can be a good indicator for future application-specific magnet setup design. The electromagnet-based mechanism shown in Fig. 5D can also be used to provide the required actuation force. As shown in Fig. 5F, we have demonstrated the feasibility of state inversion in a gripper-like array; however, a fully magnetically driven gripper demonstration is beyond the scope of the present work, as that will require a dedicated design for gripper applications.

*Supplementary conceptual note on self-actuation and remote magnetic control:* Building on the preceding discussions on electromagnetic actuation, here we propose the concept of self-actuation and remote magnetic control of the WB unit cells locally. Figure 5D shows an illustrative design of one such WB unit that can transition between its 2 stable states without application of any external mechanical force. An ultra-lightweight cylindrical tube setup containing the magnet arrangement is attached to the WB via 4 rods connected to the outer ends of the mountain creases and a rigid string attached to the central vertex of the WB. The other end of the rigid string is glued to a permanent magnet housed within the lower side of the tube between 2 stoppers. This permanent magnet is positioned such that it can move only along the *Z*-axis (i.e., the axis of the cylindrical tube). At the upper portion of the tube, a static cylindrical component made of ferromagnetic material and wrapped with a solenoid coil is integrated. This component acts as an electromagnet when an electric current flows through the coil. The direction and flow of the current (and thereby, the direction of applied force) can be controlled remotely via an electronic circuit housed on the origami module. The nearly zero-deformable facets of the WB offer suitable locations to place electronic circuit components [109]. When current flows through the coil, it induces magnetic poles in the ferromagnetic core, which then interact with the permanent magnet. If the force is repulsive, the permanent magnet moves in the negative *Z*-direction and applies a tip force via the tip-force applier to the central vertex to invert the WB’s $M_{1}$ configuration to $M_{2}$. To return to the $M_{1}$ configuration, the current direction is reversed, flipping the magnetic poles and creating an attractive force that pulls the WB’s vertex back. The overall inversion of the WB always involves deforming the structure by pulling the central vertex upward and pushing the outer vertices downward, or vice versa. This principle is ensured by the 4 rods attached to the structure. The principle is somewhat similar to the design of the support structure (WB sample holder) shown in Fig. 5E used for the snap-through testing of the magnetic actuator. Interestingly, beyond the ability to remotely and precisely (without influencing the surrounding units) control the stability states of the WB modules locally through integrated circuits, the proposed design also provides the scope of a self-sustained power source through placing solar panels in the origami facets when the proposed metasurfaces are used for space applications.

*Soft robotic untethered locomotion through sequential stability state alteration.* An array of interconnected bistable WB units, each capable of snapping between 2 stable configurations, can achieve untethered locomotion through a coordinated sequence of local transitions, actuated remotely via magnetic fields. Each unit stores elastic energy in its bistable geometry and, when triggered, rapidly snaps from one state to the other, producing a localized shape change. When this snapping is carefully sequenced across neighboring units (e.g., from rear to front), it generates a directional wave of deformation that propagates through the structure. This wave induces net movement by exploiting asymmetries in friction [110] or contact with the ground: during each snap, the structure momentarily deforms, shifting its center of mass forward while frictional anchoring prevents backward slip [111]. The magnetic actuation allows individual units to be addressed without physical contact, enabling precise temporal control over the snap-through events. As each unit snaps and resets in a programmed rhythm, the cumulative effect is a crawling or peristaltic-like motion, allowing the structure to locomote without wheels, legs, or continuous sliding surfaces, achieving true untethered, shape-driven mobility.

An embedded permanent magnet strategy can be followed for sequential actuation, wherein each bistable unit in the structure contains a small permanent magnet, typically integrated into or attached to a specific region of the unit. Locomotion can be achieved by applying an external magnetic field gradient, generated by movable permanent magnets, electromagnetic coils, or Halbach arrays, that exerts a directional force or torque on the embedded magnet. When the external field is positioned or modulated in a periodic sequence, it creates a magnetic force on the targeted unit, destabilizing its current configuration and triggering it to snap into its alternate stable state. By carefully sequencing the position and timing of these external fields, specific units can be activated one after another, producing a controlled wave of snapping transitions across the array. This enables contactless, programmable actuation of individual units, allowing the structure to achieve shape change and locomotion without any onboard power or wiring. In principle, a 2D WB array can achieve forward, backward, rotation, and any other possible motion cases depending on the algorithmic actuation control of the WB units to alter their local stability states. The simplicity of this approach makes the proposed WB arrays especially attractive for the dual functionality of shape-changing metasurface (or gripper) and untethered soft robots.

Figure 2J and Movie S6 present a schematic representation of the locomotion of a metasurface. The 2D metasurface in its unfolded flat configuration can be visualized as multiple connected strips in 2 perpendicular directions (refer to Fig. 2J, where a single such strip is highlighted). The constituting strips possess similar architectures and stability states at any point in time during locomotion. In nonflat configurations (obtainable through appropriate assignment of stability states in all the WB modules, as demonstrated in Fig. 2H), the WB metastructure can form a bidirectionally curved surface. When the metasurface initiates locomotion along a particular direction (e.g., the *X*-direction), the curvature of all the array strips along the *X*-direction is varied through state inversion of the units (refer to Fig. 2J, i). The asymmetric friction at the rear and front contact points with the rough ground can be exploited in a controlled manner (as shown in Fig. 2J) to achieve directional locomotion. Such frictional asymmetry provides an effective constraint, and the resulting reaction forces lead to motion due change of bistability states in targeted WB units. Here, an ideally rigid interunit crease connection (refer to the “Remarks on the effect of surrounding WB units” paragraph within the “Analytical modeling of elementary origami kinematics” section, i.e., $k^{j*}\to \infty$) constrains the surrounding edges of the actuated unit during state switching. It may be noted that even though the overall structure may deform due to applied external magnetic fields (global elastic deformation), there can be local bistability inversion in the WB units, wherein the connections work more like constraining boundaries. This will depend on the required inversion force that the magnetic field needs to generate, the aspect of which is investigated comprehensively in the manuscript. Since the global elastic deformation of the structure is recoverable, this component disappears when the external magnetic source is turned off, while the effect of local bistable stage switching is retained. In this context, the proposed mechanism in Fig. 5D could be more effective for local actuation and bistability control. Of course, one needs to keep in mind that our objective in the paper is to show that local control of bistability states can lead to programmable curvature, wherein applications such as robotic motion are envisaged. The general self-actuating magnet mechanism in Fig. 5D, which can be integrated to realize local state switching, can be particularly attractive for the effective control of stability states. Because this mechanism is driven by minimal electrical stimuli, individual state switches (self-contained magnetic actuators) can be controlled remotely through on-board electronics.

Similar to the *X*-direction, curvature control by state inversion of the array strip along the perpendicular (*Y*) direction enables locomotion along the *Y*-direction as well (see Fig. 2J, ii). Note that the WB units near the edges of the 2D metasurface and their state inversion play the most crucial role in achieving effective locomotion in this 2D plane. Figure 2K shows the locomotion of a gripper using the same principles as the metasurface. For example, the 1D strip of WBs (analogous to a 2-armed gripper) shown in Fig. 2K, i, moves in a single direction via sequential state inversion of the 2 end WB units. Note that this inversion can be actively controlled using an external magnetic field, as mentioned earlier concerning the discussion on far-field magneto-active control of metasurface shape and curvature. Two such strips can be attached perpendicularly to form a 4-armed gripper, as shown in Fig. 2K, ii. Sequential active inversion of the end units of a particular strip can then be used to produce crawling locomotion in the direction of that strip, and eventually, it will result in in-plane locomotion of the gripper along the desired direction.

Based on the proposed active WB arrays through introducing sequential and periodic stability state transition of selected WB units, we unravel an unprecedented class of dual functionality. It is possible to have a stationary metasurface with active modulation capability of its curvature or a soft robotic gripper (working on a particular spot), and then have untethered locomotion to move it to a different spot for performing further metasurface or gripper operations in the new location. This unprecedented multifunctional design showcases how a single active origami framework with algorithmically distributed state alteration can seamlessly transition among metasurface morphing, gripping, and locomotion tasks.

### Summary of contributions and perspective

The contribution of this article is 3-fold: (a) developing systematic analytical frameworks for characterizing the bistability of symmetric and asymmetric WB units with generic number of creases, and their magneto-active actuation; (b) proposition of 1D and 2D surface programming using primary and secondary assembly strategies that exploit algorithmic stability state inversions introducing the notion of a “digital brain”; and (c) prospective applications of the origami-based active surface curvature modulation framework in the fields of programmable meta-surfaces, conformable grippers, and robotic locomotion. In the following paragraphs, we provide further perspectives on such contributions of this article.

The primary contribution of this paper is to present a novel class of origami metasurfaces that can achieve and actively modulate surface curvature based on local and distributed control of the stability states of the constituting WB units. A range of 1D and 2D metasurfaces has been proposed, wherein uni- or bidirectional curvatures and other target shapes can be realized through far-field alteration of the WB units. For developing the 2D metasurfaces, 2 different strategies have been ideated by the continuous assembly of the WB units and a secondary grid-like assembly of multiple 1D architectures. As a special case, such secondary assembly is demonstrated as soft robotic grippers when there is just one joint in the architecture, leading to 2 or more arms. A range of analytical, numerical, and experimental investigations have been carried out systematically to demonstrate the concept of multistable origami metasurfaces and their active curvature control.

Systematic and comprehensive analysis of generic symmetric and asymmetric WB units and their bistability characteristics is scarce to find in literature. We have started the investigation with the analytical modeling of a symmetric, arbitrary number of creases in a single WB unit, which is further analyzed for the 8-crease WB modules as a special case of interest. Subsequently, the analytical model for a nonsymmetric 6-crease WB is presented. The primary objective of the analytical investigation here is to predict the motion behavior and critical inversion force for stability state alteration of a single WB module. The critical inversion force predicted by analytical models is validated with separate finite element-based numerical simulations and experimental characterization. A far-field magnetic actuation strategy (including the conceptual illustration of self-powered contactless actuation) is conceptualized for precisely controlled stability state alteration of the WB units. Based on such active WB-based building blocks, a range of 1D and 2D metasurfaces is demonstrated for achieving prescribed curvature from a flat shape, and their active modulation through far-field intelligent control.

It can be noted that more WB units in the assembly would expand the scope of attaining different on-demand curvatures and shapes, wherein a background algorithm (analogous to a “digital brain”) can decide the necessary stability states of each of the constituting WB units (i.e., building blocks). The digital brain would typically consist of an optimization algorithm with the stability state of each building block as design variables and the objective as target metasurface shapes. It should also be able to compute the necessary magnetic field for the inversion of a building block WB unit locally, depending on its surrounding stability landscape. Machine learning-based approaches can be involved for improving the computational efficiency of the digital brain, especially in the case of power-constrained scenarios like space applications and engineering scenarios with very low reaction time. It is noted in this context that the proposed piece-wise distributed bistable actuation mechanism allows a significantly higher flexibility in terms of the accuracy demand of critical inversion force compared to conventional monostable structures while targeting a specified shape. Further, the active far-field actuation strategy provides an unprecedented scope of dealing with higher weight lifting in robotic grippers or maintaining the target metasurface shape and structural integrity under unintended operational loads (such as wind or solar storm) by on-demand modulation of the inversion force through the contactless application of algorithmically controlled additional magnetic field during the operation.

Based on the proposed active WB arrays through introducing sequential and periodic stability state transition of selected WB units, we unravel an unprecedented class of multifunctionality when the proposed digital brain is further trained in the temporal domain. It is possible to have a stationary metasurface with active modulation capability of its curvature or a soft robotic gripper (working on a particular spot), and then have untethered locomotion to move it to a different spot for performing further metasurface or gripper operations in the new location.

## Conclusion

This paper exploits the bistable mechanics of WB origami units, their strategic assembly, and spatially sequential alteration of local stability states to develop a new class of active metasurfaces. While the main focus of this paper is to establish the ability of curvature modulation of a range of WB-based tessellated architectures, the scope of far-field contactless actuation for local stability state inversion of the WB units and the applications in the field of active meta-surface, conformable gripper design for irregular objects and robotic locomotions are discussed for contextualization. The unprecedented multifunctional design further showcases how a single active origami framework with algorithmically distributed state alteration can seamlessly transition among metasurface morphing, gripping, and locomotion tasks. The concept of spatially sequential and algorithmically controlled stability state alteration of WB architectures will find critical applications in the fields of active and reconfigurable antennas, aerodynamically adaptive aerofoils, soft robotics, energy-efficient grippers, biomedical apparatus, and programmable optical and acoustic devices.

## Methods

The computational and experimental approaches for demonstrating the concept of the origami metasurface are explained within Results and Discussion. Further Supplementary Materials are provided on (a) Kinematic model of six-crease asymmetric waterbomb units (WB6) (refer to Section S1), (b) Coordinates of vertices of waterbomb facets (refer to Section S2), (c) Energy-based gripping force analysis (refer to Section S3), (d) Computer-aided design of origami architectures (refer to Section S4), (e) Finite element implementation of waterbomb units (refer to Section S4), and (f) Supplementary results and visual explanations (refer to Section S6).

## Data Availability

All datasets used to generate the results are available in the main paper and the Supplementary Materials. Further details could be obtained from the corresponding author upon reasonable request.

## Supplementary Materials

Supplementary 1 Sections S1 to S6 Movies S1 to S6 Figs. S1 to S11 Tables S1 to S6

## References

[1] Koryo M. Method of packaging and deployment of large membranes in space. Inst Space Astronaut Sci Rep. 1985;(618):1–9.

[2] Sareh P, Guest SD. A framework for the symmetric generalisation of the Miura-ori. Int J Space Struct. 2015;30(2):141–152.

[3] Liu S, Lu G, Chen Y, Leong YW. Deformation of the Miura-ori patterned sheet. Int J Mech Sci. 2015;99:130–142.

[4] McPherson BN, Kauffman JL. Dynamics and estimation of origami-inspired deployable space structures: A review. In: *AIAA Scitech 2019 forum*. AIAA; 2019. p. 0480.

[5] Fraternali F, de Castro Motta J, Germano G, Babilio E, Amendola A. Mechanical response of tensegrity-origami solar modules, applications in engineering. Science. 2024;17: Article 100174.

[6] Fraternali F, Babilio E, Charandabi RN, Germano G, Luciano R, Spagnuolo G. Dynamic origami solar eyes with tensegrity architecture for energy harvesting Mashrabiyas. Appl Eng Sci. 2024;19: Article 100190.

[7] Boreanaz M, Belingardi G, Maia CDF. Application of the origami shape in the development of automotive crash box. Mater Des Proc Commun. 2020;2(4): Article e181.

[8] Du Y, Keller T, Song C, Wu L, Xiong J. Origami-inspired carbon fiber-reinforced composite sandwich materials—Fabrication and mechanical behavior. Compos Sci Technol. 2021;205: Article 108667.

[9] Li S, Fang H, Sadeghi S, Bhovad P, Wang K-W. Architected origami materials: How folding creates sophisticated mechanical properties. Adv Mater. 2019;31(5):1805282. 10.1002/adma.201805282 30516852

[10] Fonseca LM, Rodrigues GV, Savi MA. An overview of the mechanical description of origami-inspired systems and structures. Int J Mech Sci. 2022;223: Article 107316.

[11] Wang H, Zhao D, Jin Y, Wang M, Mukhopadhyay T, You Z. Modulation of multi-directional auxeticity in hybrid origami metamaterials. Appl Mater Today. 2020;20: Article 100715.

[12] Sinha A, Mukhopadhyay T. Kirigami-inspired metamaterials for programming constitutive laws: Mixed-mode multi-directional auxeticity and contact-induced stiffness modulation. iScience. 2022;25(12): Article 105656. 36590462 10.1016/j.isci.2022.105656 PMC9801249

[13] Tiwari AK, Upadhyay SH, Mukhopadhyay T. Nonlinear wave propagation and dynamic control in multi-unit kresling origami metamaterials with conical architectures. Mech Syst Signal Process. 2025;244: Article 113737.

[14] Kundu D, Ghuku S, Naskar S, Mukhopadhyay T. Extreme specific stiffness through interactive cellular networks in bi-level micro-topology architected metamaterials. Adv Eng Mater. 2023;25(8):2201407.

[15] Tiwari AK, Upadhyay SH, Mukhopadhyay T. On introducing conicity in tubular origami metastructures for programming the nonlinear dynamics in an expanded design space. J Sound Vib. 2025;603: Article 118941.

[16] Sharma A, Naskar S, Mukhopadhyay T. Programmable constitutive mode-coupling in tubular origami metamaterials. npj Metamaterials. 2025;1(1): Article 4.

[17] Wo Z, Filipov ET. Stiffening multi-stable origami tubes by outward popping of creases. Extreme Mech Lett. 2023;58: Article 101941.

[18] Miranda R, Babilio E, Singh N, Santos F, Fraternali F. Mechanics of smart origami sunscreens with energy harvesting ability. Mech Res Commun. 2020;105: Article 103503.

[19] Fang H, Chu S-CA, Xia Y, Wang K-W. Programmable self-locking origami mechanical metamaterials. Adv Mater. 2018;30(15):1706311. 10.1002/adma.201706311 29513374

[20] Xu R, Chen C, Sun J, He Y, Li X, Lu M-H, Chen Y. The design, manufacture and application of multistable mechanical metamaterials—A state-of-the-art review. Int J Extreme Manuf. 2023;5(4): Article 042013.

[21] Waitukaitis S, Menaut R, Chen BG, Van Hecke M. Origami multistability: From single vertices to metasheets. Phys Rev Lett. 2015;114(5): Article 055503. 25699454 10.1103/PhysRevLett.114.055503

[22] Mukhopadhyay T, Maheshwari P, Naskar S. Functionally graded energy-absorbing metamaterials with negative stiffness curved beams: Sequentially-adaptive modal multi-stability encoding. Proc R Soc A. 2026.

[23] Mukhopadhyay T, Adhikari S, Alu A. Probing the frequency-dependent elastic moduli of lattice materials. Acta Mater. 2019;165:654–665.

[24] Mukhopadhyay T, Naskar S, Kundu D, Adhikari S. Effective elastic moduli of space-filled multi-material composite lattices. Compos Commun. 2023;42: Article 101656.

[25] Tiwari P, Naskar S, Mukhopadhyay T. Nonlinear functionally graded metamaterials for hydrogen storage and enhanced sustainability under extreme environments. Thin-Walled Struct. 2025;210: Article 112901.

[26] Ghuku S, Sahoo S, Mukhopadhyay T. Nonlinear elasticity tailoring and failure mode manipulation of functionally graded honeycombs under large deformation. Int J Non-Linear Mech. 2025;168: Article 104935.

[27] Rodriguez-Feliciano R, Wang K-W. Synthesis of a highly programmable multistable kresling origami-inspired unit cell. Int J Mech Sci. 2024;284: Article 109768.

[28] Feng Y, Huang X, Qiu X. Bistable morphology analysis of the flexible single-vertex origami unit cell. Extreme Mech Lett. 2023;65: Article 102101.

[29] Awasthi M, Naskar S, Singh A, Mukhopadhyay T. Constitutive behavior of asymmetric multi material honeycombs with bi-level variably-thickened composite architecture. Thin-Walled Struct. 2024;203: Article 112183.

[30] Sinha P, Kundu D, Naskar S, Mukhopadhyay T. Effective elastic properties of 3d lattice materials with intrinsic stresses: Bottom-up spectral characterization and constitutive programming. Appl Math Model. 2025;140: Article 115786.

[31] Misseroni D, Pratapa PP, Liu K, Kresling B, Chen Y, Daraio C, Paulino GH. Origami engineering. Nat Rev Methods Primers. 2024;4(1):40.

[32] Sharma A, Naskar S, Mukhopadhyay T. Multi-physically programmable tubular origami meta materials: Exploitable nexus of geometry, folding mechanics and stimuli-responsive physics. Adv Sci. 2025;12(36): Article e05089. 10.1002/advs.202505089 PMC12463044 40855600

[33] Yang M, Grey SW, Scarpa F, Schenk M. Large impact of small vertex cuts on the mechanics of origami bellows. Extreme Mech Lett. 2023;60: Article 101950.

[34] Yang M, Defillion J, Scarpa F, Schenk M. Volume optimisation of multi-stable origami bellows for deployable space habitats. Acta Mech Solida Sin. 2023;36:514–530.

[35] Mitchell D. *Mathematical origami: Geometrical shapes by paper folding*. Tarquin Publications; 1997.

[36] Hanna BH. Modeling and testing of bistable waterbomb base configurations. Brigham Young University; 2014.

[37] Hanna BH, Magleby SP, Lang RJ, Howell LL. Force–deflection modeling for generalized origami waterbomb-base mechanisms. J Appl Mech. 2015;82(8): Article 081001.

[38] Chen Y, Feng H, Ma J, Peng R, You Z. Symmetric waterbomb origami. Proc Math Phys Eng Sci. 2016;472(2190):20150846. 27436963 10.1098/rspa.2015.0846 PMC4950188

[39] Rodrigues GV, Savi MA. Nonlinear dynamics and chaos of a waterbomb origami unit cell considering different symmetry conditions. Mech Res Commun. 2024;136: Article 104233.

[40] Rodrigues GV, Savi MA. Reduced-order model description of origami stent built with water bomb pattern. Int J Appl Mech. 2021;13(2):2150016.

[41] Rodrigues GV, Fonseca LM, Savi MA, Paiva A. Nonlinear dynamics of an adaptive origami-stent system. Int J Mech Sci. 2017;133:303–318.

[42] Zhang H, Zhu B, Chen B, Cui C, Li H, Zhang X. A phase diagram-based stability design method for a symmetrical origami waterbomb base. J Mech Des. 2022;144(10): Article 103303.

[43] Pratapa PP, Bellamkonda A. Thick panel origami for load-bearing deployable structures. Mech Res Commun. 2022;124: Article 103937.

[44] Mukhopadhyay T, Ma J, Feng H, Hou D, Gattas JM, Chen Y, You Z. Programmable stiffness and shape modulation in origami materials: Emergence of a distant actuation feature. Appl Mater Today. 2020;19: Article 100537.

[45] Ma J, Feng H, Chen Y, Hou D, You Z. Folding of tubular waterbomb. Research. 2020;2020: Article 1735081. 32529187 10.34133/2020/1735081 PMC7171592

[46] Grasinger M, Gillman A, Buskohl PR. Multistability, symmetry and geometric conservation in eightfold waterbomb origami. Proc R Soc A. 2022;478(2268):20220270.

[47] Boyvat M, Koh J-S, Wood RJ. Addressable wireless actuation for multijoint folding robots and devices. Sci Robot. 2017;2(8):eaan1544. 33157882 10.1126/scirobotics.aan1544

[48] Li S, Vogt DM, Rus D, Wood RJ. Fluid-driven origami-inspired artificial muscles. Proc Natl Acad Sci USA. 2017;114(50):13132–13137. 29180416 10.1073/pnas.1713450114 PMC5740677

[49] Deo A, Lee J, Gao D, Shenoy R, Haughn KP, Rong Z, Hei Y, Qiao D, Topac T, Chang F-K, et al. Super-turing synaptic resistor circuits for intelligent morphing wing. Commun Eng. 2025;4(1):109. 40523910 10.1038/s44172-025-00437-y PMC12170896

[50] Rus D, Tolley MT. Design, fabrication and control of soft robots. Nature. 2015;521(7553):467–475. 26017446 10.1038/nature14543

[51] Grey SW, Scarpa F, Schenk M. Embedded actuation for shape-adaptive origami. J Mech Des. 2021;143(8): Article 081703.

[52] Liu W, Wang C, Sun X, Guo H, Ma J, Chen Y. Origami-inspired structural design for aquatic-terrestrial amphibious robots. Adv Robot Res. 2025; Article e202500171.

[53] El-Atab N, Mishra RB, Al-Modaf F, Joharji L, Alsharif AA, Alamoudi H, Diaz M, Qaiser N, Hussain MM. Soft actuators for soft robotic applications: A review. Adv Intell Syst. 2020;2(10):2000128.

[54] Faber JA, Udani JP, Riley KS, Studart AR, Arrieta AF. Dome-patterned metamaterial sheets. Adv Sci. 2020;7(22):2001955. 10.1002/advs.202001955 PMC7675196 33240759

[55] Sharma A, Naskar S, Mukhopadhyay T. Programmable shape morphing and space deployment through graded derivatives of origami architectures. Space Sci Technol. 2025;6: Article 0363.

[56] Sinha P, Mukhopadhyay T. Programmable multi-physical mechanics of mechanical metamaterials. Mater Sci Eng R. 2023;155: Article 100745.

[57] Singh A, Mukhopadhyay T, Adhikari S, Bhattacharya B. Extreme on-demand contactless modulation of elastic properties in magnetostrictive lattices. Smart Mater Struct. 2022;31(12): Article 125005.

[58] Kundu D. Active mechanical cloaking for unsupervised damage resilience in programmable elastic metamaterials. Philos Trans R Soc A Math Phys Eng Sci. 2024;382(2278): Article 20230360. 10.1098/rsta.2023.0360 PMC11529627 39069765

[59] Mondal S, Mukhopadhyay T, Naskar S. Active heterogeneous mode coupling in bi-level multi physically architected metamaterials for temporal, on-demand and tunable programming. Commun Eng. 2025;4(1):103. 40483340 10.1038/s44172-025-00420-7 PMC12145453

[60] Leanza S, Wu S, Sun X, Qi HJ, Zhao RR. Active materials for functional origami. Adv Mater. 2024;36(9):2302066. 10.1002/adma.202302066 37120795

[61] Peraza-Hernandez EA, Hartl DJ, Malak RJ Jr, Lagoudas DC. Origami-inspired active structures: A synthesis and review. Smart Mater Struct. 2014;23(9): Article 094001.

[62] Ghuku S, Naskar S, Mukhopadhyay T. Stimuli-responsive programmable mechanics of bi-level architected nonlinear mechanical metamaterials. Mech Mater. 2025;206: Article 105333.

[63] Kundu D, Mukhopadhyay T. Pneumatic optimally tapered inflatable lattices with deployable and conformal characteristics. Space Sci Technol. 2025;5:0308.

[64] De Moura BB, Machado MR, Mukhopadhyay T. Wearable metamaterials with embodied intelligence for programmable control of human limbs tremor. Adv Intell Syst. 2026;8(3): Article e202500307.

[65] Sinha P. On-demand contactless programming of nonlinear elastic moduli in hard magnetic soft beam based broadband active lattice materials. Smart Mater Struct. 2023;32(5): Article 055021.

[66] Zaidi S, Maselli M, Laschi C, Cianchetti M. Actuation technologies for soft robot grippers and manipulators: A review. Curr Robot Rep. 2021;2(3):355–369.

[67] Tawk C, Spinks GM, in het Panhuis M, Alici G. 3D printable linear soft vacuum actuators: Their modeling, performance quantification and application in soft robotic systems. IEEE/ASME Trans Mechatron. 2019;24(5):2118–2129.

[68] Li S, Stampfli JJ, Xu HJ, Malkin E, Diaz EV, Rus D, Wood RJ. A vacuum-driven origami “magic-ball” soft gripper. In:2019 International Conference on Robotics and Automation (ICRA) IEEE; 2019. p. 7401–7408.

[69] Melancon D, Forte AE, Kamp LM, Gorissen B, Bertoldi K. Inflatable origami: Multimodal deformation via multistability. Adv Funct Mater. 2022;32(35):2201891.

[70] Lee J-Y, Kang BB, Lee D-Y, Baek S-M, Kim W-B, Choi W-Y, Song J-R, Joo H-J, Park D, Cho K-J. Development of a multi-functional soft robot (SNUMAX) and performance in robosoft grand challenge. Front Robot AI. 2016;3:63.

[71] Xiang Z, Hongwei L, Bingxiao D, Lu C, Xiaoqian C, Yiyong H. Design and experimental validation of a cable-driven continuum manipulator and soft gripper. In:2019 IEEE International Conference on Robotics and Biomimetics (ROBIO) IEEE; 2019. p. 1965–1968.

[72] Treml B, Gillman A, Buskohl P, Vaia R. Origami mechanologic. Proc Natl Acad Sci USA. 2018;115(27):6916–6921. 29915077 10.1073/pnas.1805122115 PMC6142273

[73] Chung H-J, Parsons AM, Zheng L. Magnetically controlled soft robotics utilizing elastomers and gels in actuation: A review. Adv Intell Syst. 2021;3(3):2000186.

[74] Hu W, Lum GZ, Mastrangeli M, Sitti M. Small-scale soft-bodied robot with multimodal locomotion. Nature. 2018;554(7690):81–85. 29364873 10.1038/nature25443

[75] Wu S, Hamel CM, Ze Q, Yang F, Qi HJ, Zhao R. Evolutionary algorithm-guided voxel encoding printing of functional hard-magnetic soft active materials. Adv Intell Syst. 2020;2(8):2000060.

[76] Kim Y, Parada GA, Liu S, Zhao X. Ferromagnetic soft continuum robots. Sci Robot. 2019;4(33):eaax7329. 33137788 10.1126/scirobotics.aax7329

[77] Kitano S, Komatsuzaki T, Suzuki I, Nogawa M, Naito H, Tanaka S. Development of a rigidity tunable flexible joint using magneto-rheological compounds—Toward a multijoint manipulator for laparoscopic surgery. Front Robot AI. 2020;7:59. 33501227 10.3389/frobt.2020.00059 PMC7805682

[78] Bowen L, Springsteen K, Feldstein H, Frecker M, Simpson TW, von Lockette P. Development and validation of a dynamic model of magneto-active elastomer actuation of the origami waterbomb base. J Mech Robot. 2015;7(1): Article 011010.

[79] Bowen L, Springsteen K, Frecker M, Simpson T. Trade space exploration of magnetically actuated origami mechanisms. J Mech Robot. 2016;8(3): Article 031012.

[80] Cui C, Zhang X, Zhu B, Li H, Zhang H, Wang R, Lai J, Feng K. A novel analysis method for magnetically actuated soft origami mechanisms. Mech Mach Theory. 2023;186: Article 105353.

[81] Hanna BH, Lund JM, Lang RJ, Magleby SP, Howell LL. Waterbomb base: A symmetric single-vertex bistable origami mechanism. Smart Mater Struct. 2014;23: Article 094009.

[82] Flores J, Stein-Montalvo L, Adriaenssens S. Effect of crease curvature on the bistability of the origami waterbomb base. Extreme Mech Lett. 2022;57: Article 101909.

[83] Zhai Z, Wu L, Jiang H. Mechanical metamaterials based on origami and kirigami. Appl Phys Rev. 2021;8(4): Article 041319.

[84] Gillman A, Wilson G, Fuchi K, Hartl D, Pankonien A, Buskohl P. Design of soft origami mechanisms with targeted symmetries. Actuators. 2018;8(1):3.

[85] Chen Y, Yan J, Feng J. Geometric and kinematic analyses and novel characteristics of origami inspired structures. Symmetry. 2019;11(9):1101.

[86] Lee Y-K, Kim J, Lien J-M, Lee Y-J, Choi I-S. Recent progress in shape-transformable materials and their applications. Electron Mater Lett. 2022;18(3):215–231.

[87] Callens SJ, Zadpoor AA. From flat sheets to curved geometries: Origami and kirigami approaches. Mater Today. 2018;21(3):241–264.

[88] Zhai Z, Wang Y, Lin K, Wu L, Jiang H. In situ stiffness manipulation using elegant curved origami. Sci Adv. 2020;6(47):eabe2000. 33208377 10.1126/sciadv.abe2000 PMC7673814

[89] Chen Z, Tighe B, Zhao J. Origami-inspired modules enable a reconfigurable robot with programmable shapes and motions. IEEE/ASME Trans Mechatron. 2022;27(4):2016–2025.

[90] Park Y, Kang J, Na Y. Reconfigurable shape morphing with origami-inspired pneumatic blocks. IEEE Rob Autom Lett. 2022;7(4):9453–9460.

[91] Yao S, Liu X, Georgakopoulos SV. Morphing origami conical spiral antenna based on the nojima wrap. IEEE Trans Antennas Propag. 2017;65(5):2222–2232.

[92] Nathan D, Deo A, Haughn K, Yi S, Lee J, Gao D, Shenoy R, Xu M, Tran IC, Zheng J-G, et al. Si-based self-programming neuromorphic integrated circuits for intelligent morphing wings. J Compos Mater. 2022;56(30):4561–4575.

[93] Haughn KP, Gamble LL, Inman DJ. Deep reinforcement learning achieves multifunctional morphing airfoil control. J Compos Mater. 2023;57(4):721–736.

[94] Dudek KK, Kadic M, Coulais C, Bertoldi K. Shape-morphing metamaterials. Nat Rev Mater. 2025;10(10):783–798.

[95] Dudek KK, Duncan O, Martínez JAI, Kadic M. Mechanical metamaterial-based structure with magnetically controlled nonreversibility and nonreciprocity for programmable locomotion. Adv Sci. 2025;12(32): Article e03088. 10.1002/advs.202503088 PMC12407254 40538179

[96] Fei Z, Xu D, Zhao Y, Han Z, Song L, Ma R, Guo Y. From the Yoshimura origami pattern to foldable structures: Exploration of crease design. Thin-Walled Struct. 2025;209: Article 112888.

[97] Cai J, Deng X, Xu Y, Feng J. Motion analysis of a foldable barrel vault based on regular and irregular Yoshimura origami. J Mech Robot. 2016;8(2): Article 021017.

[98] Hernandez EP, Hartl DJ, Lagoudas DC. Active origami. *Active Origami*. Springer; 2019.

[99] Meyer P, Finder J, Hühne C. Test methods for the mechanical characterization of flexure hinges. Exp Mech. 2023;63(7):1203–1222.

[100] Feng Y, Wang M, Qiu X. A simplified mechanical model of the crease in the flexible origami structures. Int J Solids Struct. 2022;241: Article 111530.

[101] Grey SW, Scarpa F, Schenk M. Mechanics of paper-folded origami: A cautionary tale. Mech Res Commun. 2020;107: Article 103540.

[102] Torsion springs explained: Essential guide for innovators and manufacturers. 2025. [accessed 17 May 2025] https://www.thespringstore.com/torsion-springs.html

[103] Zhou H, Fang H, Wu H, Xu J. Effects of inter-cell connections on the multi-stable dynamics of dual-cell stacked Miura-origami structures. J Sound Vib. 2023;553: Article 117682.

[104] Baharisangari N, Li S. Exploiting the asymmetric energy barrier in multi-stable origami to enable mechanical diode behavior in compression. In:International Design Engineering Technical Conferences and Computers and Information in Engineering Conference. 59247 American Society of Mechanical Engineers; 2019. p. V05BT07A029.

[105] Hasan MR, Davies IJ, Pramanik A, John M, Biswas WK. Potential of recycled PLA in 3D printing: A review. Sustain Manuf Serv Econ. 2024;3: Article 100020.

[106] Tan X, Chen S, Wang B, Zhu S, Wu L, Sun Y. Design, fabrication, and characterization of multistable mechanical metamaterials for trapping energy. Extreme Mechanics Letters. 2019;28:8–21.

[107] Aghamiri S, Sedaghati R. Magnetoactive metamaterials: A state-of-the-art review. Adv Eng Mater. 2025;27(23): Article e202501312.

[108] Bushman BB. Apparatus and method for amplifying a magnetic beam. US Patent: 5,929,732. 1999.

[109] Johnson K, Arroyos V, Ferran A, Villanueva R, Yin D, Elberier T, Aliseda A, Fuller S, Iyer V, Gollakota S. Solar-powered shape-changing origami microfliers. Sci Robot. 2023;8(82):eadg4276. 37703382 10.1126/scirobotics.adg4276

[110] Farhadi D, Pernigoni L, Melancon D, Bertoldi K. Origami crawlers: Exploring a single origami vertex for complex path navigation. Adv Mater. 2025;37(34): Article 2502293. 10.1002/adma.202502293 40492896

[111] Ze Q, Wu S, Nishikawa J, Dai J, Sun Y, Leanza S, Zemelka C, Novelino LS, Paulino GH, Zhao RR. Soft robotic origami crawler. Sci Adv. 2022;8(13):eabm7834. 35353556 10.1126/sciadv.abm7834 PMC8967224

