# Supplementary material for: Programmable Deployment of Multistable Origami Metasurfaces through Algorithmically Distributed State Alteration
Source: Research (Wash D C). 2026 Aug 18;9:1287. doi: 10.34133/research.1287 (PMC13481790; doi:10.34133/research.1287)
Supplement: Supplementary file 1 [file research.1287.f1.zip › [Cleaned_v2] Supplimentary_Manuscript.pdf]

Supplementary material

Programmable Deployment of Multi-stable Origami Meta-surfaces through Algorithmically Distributed State Alteration

S. Mondal[a], T. Mukhopadhyay[a], S. Naskar[a,*]

[a] *School of Engineering, University of Southampton, Southampton, UK*

Contents

S1 Kinematic model of six-crease asymmetric waterbomb units (WB6) 2

S2 Coordinates of vertices of waterbomb facets 4

S2.1 Coordinate parameters for WB6 unit 4

S2.2 Coordinate parameters for WB8 unit 5

S3 Energy-based gripping force analysis 6

S4 Computer-aided design of origami architectures 8

S5 Finite element implementation of waterbomb units 9

S6 Supplementary results and visual explanations 11

Supplementary movies

Movie S1: Experimental demonstration of Figure S10(B).

Movie S2: Experimental demonstration of Figure S10(H).

Movie S3: Experimental demonstration of Figure 5(E).

Movie S4: Experimental demonstration of Figure 5(F)(d).

Movie S5: Experimental demonstration of two fabrication techniques for PLA-based WB8 units.

Movie S6: Conceptual illustration of crawling locomotion of metasurfaces, as described in Figure 2(J).

*Email address: S.Naskar@soton.ac.uk (SN)

S1. Kinematic model of six-crease asymmetric waterbomb units (WB6)

The present formulation is derived following two ways. Firstly, an arbitrary configuration from the first stable state (M_1) is considered to form the 3D-geometry-based kinematic model for the same configuration. The same approach is later implemented with M_2 configuration as well. The two primary geometric parameters associated with the present unit cell are the side length (s) and θ, the angle between the valley crease and negative $Z-$axis. However, the other angle θ_f is also required, especially to describe the second stable configuration, which can be defined as the angle between the negative $Z-$ axis and the larger triangular facets $\triangle AED$ (or $\triangle ABG$). The configurations, associated with M_1 conform to the condition: $0° < \theta < 90°$, where the condition for the configurations associated with M_2 can be given as $90° < \theta_f < 180°$. Based on the whole folding process, the following boundary conditions can be deduced: 1) B, D, E, G are restrained along the z direction, and 2) the vertex A is displaceable vertically along $Z-$axis only.

Based on these two conditions, the coordinates of the configuration (refer to Figure S1(B)) for all the seven nodes can be calculated as follows

$$A \equiv (0, 0, z) \tag{S1a}$$

$$B \equiv (x_1, s, 0) \tag{S1b}$$

$$C \equiv (0, y_2, z_2) \tag{S1c}$$

$$D \equiv (-x_1, s, 0) \tag{S1d}$$

$$E \equiv (-x_1, -s, 0) \tag{S1e}$$

$$F \equiv (0, -y_2, z_2) \tag{S1f}$$

$$G \equiv (x_1, -s, 0) \tag{S1g}$$

where x_1, y_2 and z_2 are the displacement of the associated nodes along X, Y and Z-axis from its flat-planar configuration. Because of the symmetry about the $Y - Z$ plane, x_2 will be the only x-displacement of all four in-plane nodes B, D, E, and G, whereas ($|y_2, z_2|$) will be similar for both the end nodes (C, F) of the valley. Applying length constraints on the edges of $\triangle ABC$, we get $\overline{AB} = s\sqrt{2}$, and $\overline{AC} = s$.

$$\overline{AB} = s\sqrt{2} \quad \Longrightarrow \quad x_1^2 + s^2 + z^2 = 2s^2 \quad \Longrightarrow \quad x_1 = \sqrt{s^2 - z^2}, \tag{S2}$$

$$\text{Also,} \quad \overline{AC} = s \quad \Longrightarrow \quad y_2^2 + (z - z_2)^2 = s^2 \tag{S3}$$

Now, applying another angular constraint $\angle BAC = \pi/4$ at all the folding instants, we can derive the

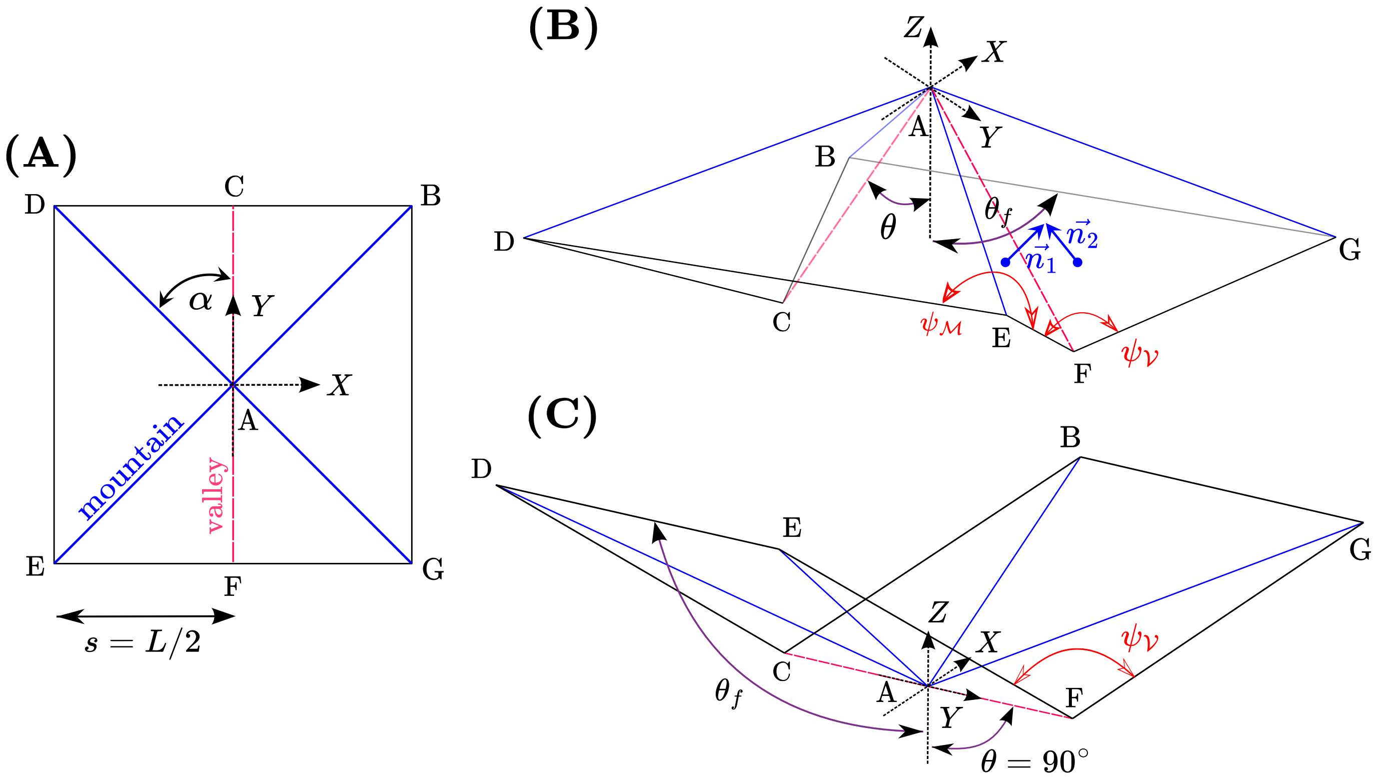

Fig. S1: Geometry of single six-crease waterbomb (WB) unit. (A) Flat unfolded planar configuration of WB. **(B)** First stable (M_1) configuration of WB. **(C)** Second stable (M_2) configuration of WB. Note that the origin of the primary coordinate system $X - Y - Z$ is placed at the vertex in-plane with the flat-planar configuration. L denotes the length of the squared flat-waterbomb base.

second condition as follows

$$\angle BAC = \alpha = \pi/4 \implies \frac{y_2 \cdot s + z \cdot (z - z_2)}{s\sqrt{2} \cdot s} = \frac{1}{\sqrt{2}} \implies z^2 - zz_2 + sy_2 = s^2 \tag{S4}$$

where $\overline{BA}$ and $\overline{CA}$ are taken as line vectors: $\vec{BA} = x_1\hat{i} + s\hat{j} - z\hat{k}$, $\vec{CA} = y_2\hat{j} + (z_2 - z)\hat{k}$. Based on the two constraints (refer to equations (S3) and (S4)), we can deduce a relation between y_2, s, and z as follows

$$y_2 = \frac{s(s^2 \pm z^2)}{(s^2 + z^2)} \implies y_2 = \frac{s(s^2 - z^2)}{(s^2 + z^2)} \quad (y_2 \text{ is varying in nature}) \tag{S5}$$

Substituting equation (S5) in equation (S3), we get

$$z_2 = \frac{-z(s^2 - z^2)}{(s^2 + z^2)} \tag{S6}$$

Now observing the motion of the valley fold $\overline{AC}$, the angle θ can be related to s as, $\sin\theta = y_2/s$. Based on this, equations (S5) and (S6) can be rewritten as follows

$$z^2 = s^2\frac{(1 - \sin\theta)}{(1 + \sin\theta)} \tag{S7}$$

$$z_2 = -z\sin\theta \tag{S8}$$

Finally, the dihedral angle, $\psi_{\mathcal{V}}$ can be calculated from the angle between normal vectors of the plane $\triangle ABC$ and $\triangle ACD$. Let's take the normal of plane $\triangle ABC$ as $\vec{n_1}$, whereas the same for $\triangle ACD$ as $\vec{n_2}$. These two can be given mathematically as

$$\vec{n_1} = \frac{\vec{BC} \times \vec{AC}}{|BC||AC|} \tag{S9}$$

$$\vec{n_2} = \frac{\vec{DC} \times \vec{AC}}{|DC||AC|} \tag{S10}$$

where $\vec{BC}$, $\vec{AC}$ and $\vec{DC}$ can be calculated from equation (S1). For instance, $\vec{BC} = \hat{i}(-x_1) + \hat{j}(y_2 - s) + \hat{k}(z_2)$, $\vec{AC} = \hat{j}(y_2) + \hat{k}(z_2 - z)$ and $|BC| = |AC| = |DC| = s$. After substitution of the derived expressions of x_1, y_2, z_2 and z (refer to equations S2, S7, S8, S5) and algebraic simplification, the following expressions for $\vec{n_1}$ and $\vec{n_2}$ can be given as

$$\vec{n_1} = \frac{1}{s^2}\left(sz\hat{i} - \left(z(1+\sin\theta)\sqrt{s^2-z^2}\right)\hat{j} - \left(s\sin\theta\sqrt{s^2-z^2}\right)\hat{k}\right) \tag{S11}$$

$$\vec{n_2} = \frac{1}{s^2}\left(sz\hat{i} + \left(z(1+\sin\theta)\sqrt{s^2-z^2}\right)\hat{j} + \left(s\sin\theta\sqrt{s^2-z^2}\right)\hat{k}\right) \tag{S12}$$

The explicit expression for the angle $\psi_{\mathcal{V}}$ can be given by

$$\psi_{\mathcal{V}} = \cos^{-1}\left(\vec{n_1} \cdot \vec{n_2}\right) = \cos^{-1}\left(\frac{1-3\sin\theta}{1+\sin\theta}\right) \tag{S13}$$

Similarly, $\psi_{\mathcal{M}}$ can be found in an explicit form as follows

$$\psi_{\mathcal{M}} = \pi + \cos^{-1}\left(2\sin\theta - 1\right) \tag{S14}$$

Now coming to the second stable configuration (refer to Figure S1(C)), the dihedral angles can be derived without diving much into the algebraic trigonometric operations, rather simply from the geometry. The expressions for the same are as follows

$$\psi_{\mathcal{M}} = 0 \tag{S15}$$

$$\psi_{\mathcal{V}} = 2\left(\pi - \theta_f\right) \tag{S16}$$

S2. Coordinates of vertices of waterbomb facets

Based on simple geometric analysis of WB units in the coordinate system shown in Figure S2, the coordinate parameters (x^i_j, y^i_j, z^i_j) (refer to equation (10)) can be obtained. Note that here the index i refers to the ith triangular facet of the corresponding WB unit.

S2.1. Coordinate parameters for WB6 unit

In case of WB6 units, the angle θ_f refers to the angle made by one triangular rigid facet with the negative Z-axis. It can be related to the valley line angle θ for the given configuration shown in

Table S1: Coordinate parameters for all six facets of WB6 units

	$i=1$	$i=2$	$i=3$	$i=4$	$i=5$	$i=6$
x_2^i	$\frac{L}{2}\sin\theta_f$	x_3^1	x_3^2	x_3^3	x_3^4	x_3^5
y_2^i	$-\frac{L}{2}$	y_3^1	y_3^2	y_3^3	y_3^4	y_3^5
z_2^i	$-\frac{L}{2}\cos\theta_f$	z_3^1	z_3^2	z_3^3	z_3^4	z_3^5
x_3^i	$\frac{L}{2}\sin\theta_f$	0	$-\frac{L}{2}\sin\theta_f$	$-\frac{L}{2}\sin\theta_f$	0	x_2^1
y_3^i	$\frac{L}{2}$	$\frac{L}{2}\frac{\sin^2\theta_f}{1+\cos^2\theta_f}$	$\frac{L}{2}$	$-\frac{L}{2}$	$-\frac{L}{2}\frac{\sin^2\theta_f}{1+\cos^2\theta_f}$	y_2^1
z_3^i	$-\frac{L}{2}\cos\theta_f$	$-L\frac{\cos\theta_f}{1+\cos^2\theta_f}$	$-\frac{L}{2}\cos\theta_f$	$-\frac{L}{2}\cos\theta_f$	$-L\frac{\cos\theta_f}{1+\cos^2\theta_f}$	z_2^1

Table S2: Coordinate parameters for all eight facets of WB8 units

	$i=1$	$i=2$	$i=3$	$i=4$	$i=5$	$i=6$	$i=7$	$i=8$
x_2^i	$\frac{L\sin\tau}{2}$	x_3^1	x_3^2	x_3^3	x_3^4	x_3^5	x_3^6	x_3^7
y_2^i	$-\frac{L\sin\tau}{2}$	y_3^1	y_3^2	y_3^3	y_3^4	y_3^5	y_3^6	y_3^7
z_2^i	$-\frac{L\cos\tau}{\sqrt{2}}$	z_3^1	z_3^2	z_3^3	z_3^4	z_3^5	z_3^6	z_3^7
x_3^i	$\frac{L\sin\theta}{2}$	$\frac{L\sin\tau}{2}$	0	$\frac{-L\sin\tau}{2}$	$\frac{-L\sin\theta}{2}$	$\frac{-L\sin\tau}{2}$	0	x_2^1
y_3^i	0	$\frac{L\sin\tau}{2}$	$\frac{L\sin\theta}{2}$	$\frac{L\sin\tau}{2}$	0	$\frac{-L\sin\tau}{2}$	$\frac{-L\sin\theta}{2}$	y_2^1
z_3^i	$-\frac{L\cos\theta}{2}$	$-\frac{L\cos\tau}{\sqrt{2}}$	$-\frac{L\cos\theta}{2}$	$\frac{-L\cos\tau}{\sqrt{2}}$	$\frac{-L\cos\theta}{2}$	$\frac{-L\cos\tau}{\sqrt{2}}$	$\frac{-L\cos\theta}{2}$	z_2^1

Figure S2(B) by the following expression.

$$\theta_f = -\sin^{-1}\left(\frac{1-\cos^2\theta}{\cos^2\theta+1}\right) \tag{S17}$$

S2.2. Coordinate parameters for WB8 unit

In case of WB8 units, the angle τ refers to the angle made by the mountain fold with the negative Z axis. It can be expressed in terms of the angle θ for the given folded configuration of WB8, shown in Figure S2, by the following expression [1].

$$\tau = \frac{\pi}{2} - \tan^{-1}\left(\frac{\cos\theta(\sqrt{2}-\sin\theta)}{\sqrt{2}\cos^2\theta+\sin\theta}\right) \tag{S18}$$

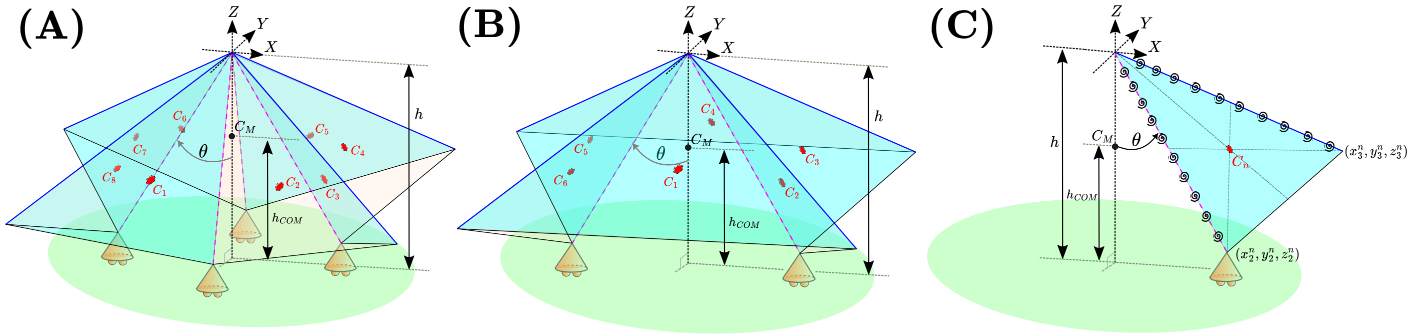

Fig. S2: **(A, B)** Visualization of centres of mass in waterbomb units with (A) eight creases (WB8), and (B) six creases (WB6). **(C)** The centre of mass for individual facets of the waterbombs and associated two torsional spring equivalent creases are depicted. C_M and C_n ($n \in \mathbb{N}$) denote the centre of mass of the waterbomb unit and of its individual facet, respectively. h_{COM} and h are the vertical distances of C_M and the centre vertex from its flat planar configuration at the given folded configuration θ. Note that $h = (L/2)\cos\theta$, where the waterbomb unit is formed from a square shaped plane sheet of side length L. Here the blue-colored solid lines and magenta-colored dashed lines represent the mountain and valley folds, respectively.

S3. Energy-based gripping force analysis

We would like to emphasize that the focus of the present paper is to propose a framework for modulating the geometry of origami-architected 1D and 2D surfaces, where metasurface, gripper and robotic crawling are presented as potential applications. In other words, the gripper is shown here primarily as a proof-of-concept application of the programmable sequential state-alteration strategy developed in the present work, rather than as a dedicated study of force-optimized grasping. However, for the sake of completeness, we have provided further insights here concerning gripping force.

Based on the kinematic and energy descriptions similar to the present paper, together with the further geometric and kinematic formulations, the gripping force is expected to depend on (1) the number of origami units in the gripper arm, which determines the overall compliance and achievable curvature for a given configuration of inverted states, (2) the geometric parameters and crease stiffness of each unit, which govern the unit-level resisting moment and stored elastic energy during switching, and therefore influence the contact force transmitted to the grasped object. To clarify this dependence while maintaining the manuscript's focus on programmable deployment, we have given here a general energy explanation to rationalize the force trends.

Let θ denotes the primary folding variable (like the single degree of freedom angle, considered in the main paper section on *analytical modelling of elementary origami kinematics*) of the waterbomb unit and its assembly. The elastic strain energy stored in the units' creases during folding is written as $U_{el}(\theta)$. In addition, the embedded magnet interacts with the external magnetic field, contributing a

magnetic potential energy

$$U_{\text{mag}}(\theta) = -\mathbf{m}(\theta) \cdot \mathbf{B}(\theta)$$

where $\mathbf{m}$ is the magnetic dipole moment of the embedded magnet-setup and $\mathbf{B}$ is the externally applied magnetic field. As the unit folds, the position and orientation of the magnets vary with θ, so the magnetic interaction energy becomes an explicit function of the folding variable. The generalized moments (torque [2]) associated with folding are then obtained from the energy gradients:

$$M_{\text{mag}}(\theta) = \frac{\partial U_{\text{mag}}}{\partial \theta}, \qquad M_{\text{el}}(\theta) = \frac{\partial U_{\text{el}}}{\partial \theta}.$$

Under quasi-static conditions, equilibrium requires

$$M_{el}(\theta) = M_{mag}(\theta) \tag{S19}$$

and switching between bistable states occurs when the magnetic actuation can overcome the maximum resisting elastic moment (equivalently, the elastic energy barrier):

$$max\, M_{\text{el}}(\theta) \;\leq\; max\, M_{\text{mag}}(\theta) \tag{S20}$$

Or,

$$\Delta U_{\text{el}} \;\leq\; \Delta U_{\text{mag}}. \tag{S21}$$

Using the geometric mapping between θ and the unit's configuration (similar to the one provided in Section S1 for WB6), the end-effector displacement (or curvature) of a one-dimensional array becomes a function of the set of local folding states. Now, for a gripper-object indentation x, the contact force follows from virtual work:

$$F \;=\; \frac{\partial U_{\text{tot}}}{\partial x} \;=\; \frac{\partial U_{\text{tot}}}{\partial \theta}\,\frac{\partial \theta}{\partial x}$$

where the total generalized potential is $U_{total} = U_{el} - U_{mag}$. Substituting the moment expressions yields

$$F \;=\; \frac{\partial U_{\text{tot}}}{\partial \theta}\,\frac{\partial \theta}{\partial x} \;=\; \Big(M_{\text{mag}}(\theta) - M_{\text{el}}(\theta)\Big)\,\frac{\partial \theta}{\partial x},$$

This expression makes the governing dependencies explicit as follows: (1) Influence of the unit's crease stiffness and geometry. Increasing crease stiffness increases $\frac{\partial U_{el}}{\partial \theta}$, thereby increasing the resisting moment and the available contact force once the unit is constrained by an object. Geometric parameters likewise modify the energy landscape and moment-angle relation [3]. (2) Influence of the number of units. Changing the number of units alters the kinematic transmission factor $\frac{\partial \theta}{\partial x}$, which reflects load sharing and overall compliance of the array. A larger number of units generally increases global compliance, redistributing deformation and modifying the effective force response at the end effector.

It is worthy to note that the experimentally measured gripping force also depends on the geometry,

weight of the grasped object and the associated contact and friction conditions. For instance, while grabbing an object, the contact force (F_{con}) between the gripper and the object can be broken down broadly into two components: a normal contact force component (F_{normal}) and a frictional force component ($F_{friction} = \mu F_{normal}$). Theoretically, the sum of normal contact forces generated by all gripping fingers tends to zero while the sum of frictional forces should be equal to or greater than the object's weight [4]. The coefficient of friction between fingertips and objects plays a crucial role in the grasping process, and a larger coefficient leads to a higher maximum grasping weight. In this context, F_{normal} can directly be correlated to the afore-derived gripper-object indentation force F.

Further, to the analytical insights presented here, a comprehensive experimental characterization of gripping force as a function of unit number, geometry, and object properties is identified as an important direction for future work.

S4. Computer-aided design of origami architectures

Robust models for a single waterbomb unit are developed such that they accurately resemble the geometrical behaviour and are able to demonstrate the inversion between the two bistable states. The purpose of the model is to function as a building block, which could be imported into a higher-level assembly used to form an array of multiple waterbombs.

The WB8 model is constructed from eight identical facets, which are defined parametrically using equations, such that the ratio of the two short sides of the facet can be varied. The method used for connecting adjacent facets to one another to form the waterbomb structure is applying coincident mate feature to the aligned edges and corner vertices of connected facets [5]. This allowed the facets to rotate around one another at the edges, modelling the folding behaviour of the creases. The WB6 model is assembled similarly, with a second larger facet also produced for the model. The most important behaviour which the single waterbomb unit models demonstrate is the ability to switch between the two well-defined bistable states (M_1 and M_2) along with the ability to modify the rest angles in each of those states. Note here that the initial stable dihedral angle between the facets is referred to as the rest angle. The models made use of configurations in SolidWorks to switch between the bistable states. This is achieved by introducing two waterbomb models into the assembly, each arranged into one of the bistable states. The design table feature is used to suppress the relevant mates and components, such that only one state is present in the assembly at any one time [6]. For the WB8 models, eight new facets are introduced, and for the WB6 model, six new facets are introduced. This allows any top-level mates applied to the waterbomb assembly to function as intended, irrespective of the existing stability state.

These facets are mated to the unsuppressed waterbomb in the assembly. Which waterbomb the facets are mated to is dependent on the configuration selected, and hence, certain mates are again suppressed depending on the inversion state using the design table. The original facets are then hidden to avoid accidental mating. Figure S7(A) shows the two possible configurations (M_1 and M_2) for WB8 model, which are controlled by the suppression state of the various mates and components in the design table. The most reliable method to fix the waterbombs rest angle is to fix ψ_v (dihedral angle of valley). This is achieved using the angle mate feature in SolidWorks. The presence of an edge mate between the adjacent facets allows the edge to be used as fixing ψ_v, preferable as opposed to fixing ψ_m or θ. The values of ψ_v used for the angle mate are added to the design table. This means that additional configurations can be added with independently varying ψ_v values for each of the sides of the waterbomb.

The local control of the bi-stable states is crucial in the current study to achieve targeted shapes. Each deformation mode (i.e. stable states) is set up as a separate configuration of the waterbomb unit subassembly, such that the deformation mode of each waterbombs is controlled by a design table in the array assembly. The design table in the array assembly is set up to allow the inversion state of each waterbomb in the array to be controlled independently, with if-statement formulas in the design table to control which deformation mode each waterbomb should have based on its inversion state and the connected waterbomb units. The overall curvature of the assembly is obtained based on the stability states of individual constituting building block waterbomb units.

S5. Finite element implementation of waterbomb units

As part of the numerical analysis, a finite element analysis (FEA) is performed on the symmetric WB8 models. This analysis is carried out to compare and validate both the analytical results and the experimental data (refer to Figure S10(C)). Within COMSOL, multi-body physics is used as the physics engine using a static solver. The models are created in SolidWorks to allow for complete integration between the creases and facets. The models are created using the vertices coordinate points from MATLAB and modified to incorporate the crease width. These points are taken as the mid-surface of the facets and creases when the models are extruded. The models are then imported into COMSOL and attached through a union of the geometries. For the nodes B and F, the displacements are taken as $u_y = u_z = 0$, whereas for the nodes D and H, the same is $u_x = u_z = 0$. For the meshing of the structure, constant sizing is used throughout the different models and a tetrahedral (tets) discretization scheme is maintained. A mesh convergence study is performed using COMSOL's in-built mesh sizing from the physics of the analysis. This allows an optimal mesh to be used considering the simulation

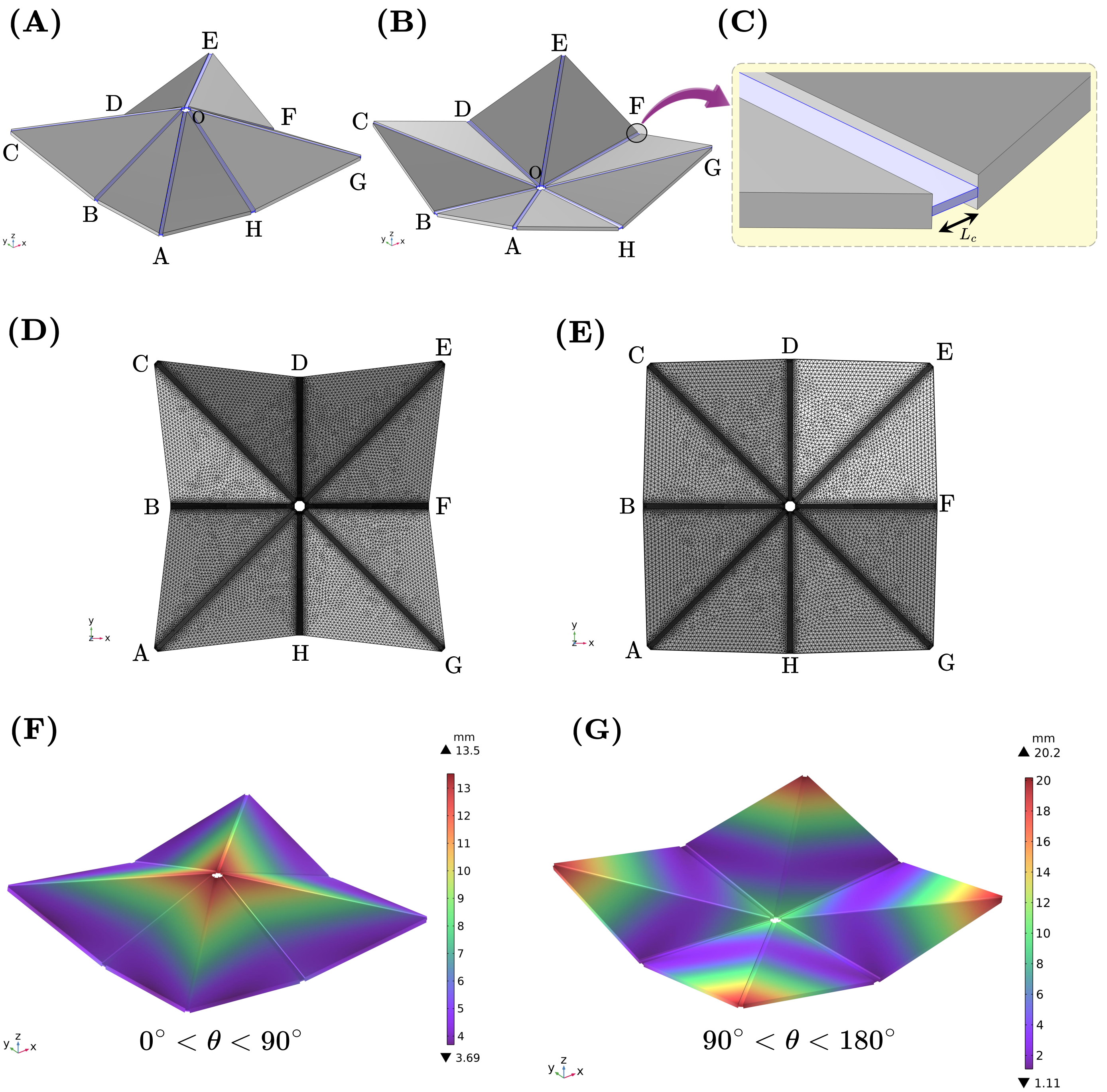

Fig. S3: Finite element implementation of waterbomb units. **(A)** CAD geometry of waterbomb in M_1 state. **(B)** CAD geometry of waterbomb in M_2 state. **(C)** Zoomed view of individual crease of length L_c. Note that the creases are taken as SLFP (small-length flexural pivot). **(D)** Meshed model of waterbomb in M_1 state. **(E)** Meshed model of waterbomb in M_2 state. Note that tetrahedral (tets) mesh elements are used to discretize the models here. **(F)** Finite element displacement plot for WB8 in M_1 state ($0^\circ < \theta < 90^\circ$). **(G)** Finite element displacement plot for WB8 in M_2 state ($90^\circ < \theta < 180^\circ$).

time, equipment limitations and the percentage discrepancies to analytical models. For instance, for M_1 model, shown in Figure S3(D), a mesh with the sizing between 2×10^{-3} and 2×10^{-5} for the maximum and minimum element size respectively is utilised. This allows smaller elements around the contact

points between the crease and facet.

The force is applied and the displacement of the centre vertex is recorded for all models. The force is applied to the model until it reaches the neutral state. Investigations are completed to try and get the model to go from one state to another; however, as the model passes the neutral state it starts to deform due to the conditions placed upon it. Therefore, the model is displaced only upto the neutral state with the forces required for both starting states (M_1 and M_2) which could then be compared. Finally, the displacement recorded from the analysis is converted into the input angle at the given applied force. This is then plotted as shown in Figure S10(C).

S6. Supplementary results and visual explanations

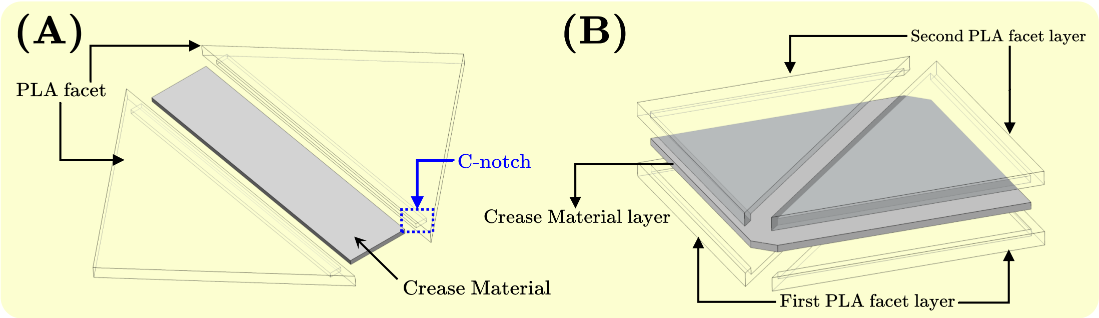

Fig. S4: Two fabrication techniques for attaching 3D printed PLA based facets with crease material. (A) C-Section based notch. **(B)** Stop-Start (SS) printing technique. Note that these two techniques in case of WB8 fabrication are also shown in Supplementary Movie S5.

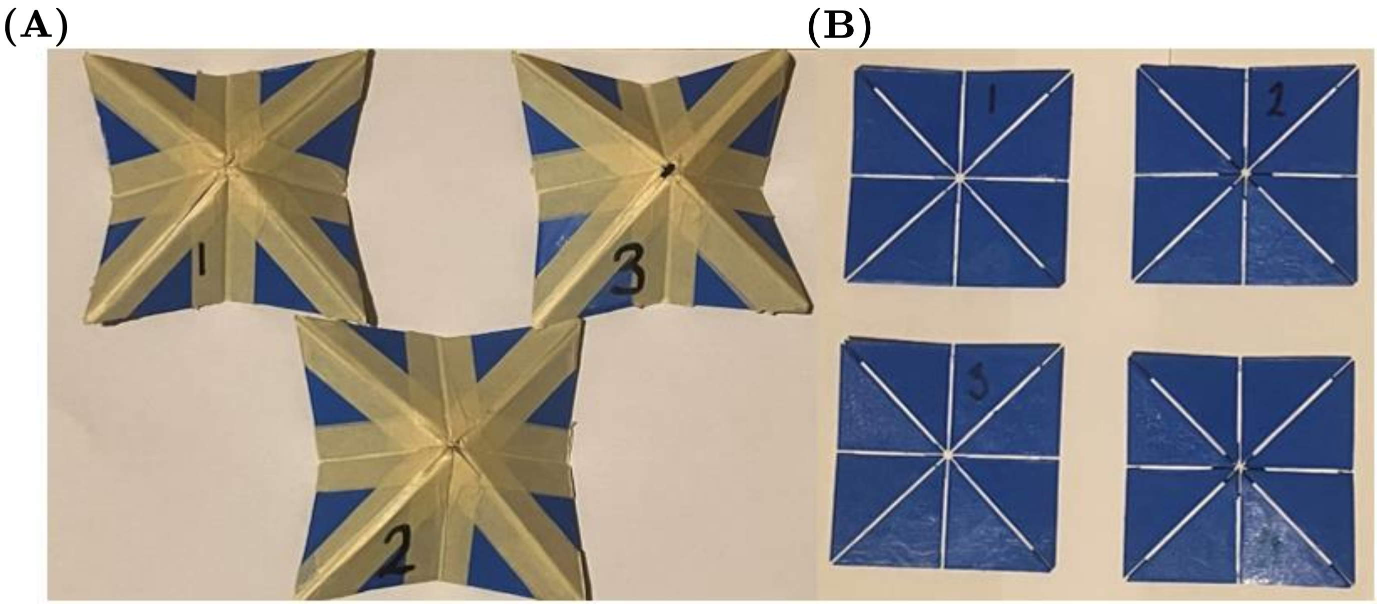

Fig. S5: Waterbomb module prototypes for testing (A) Double-layered Masking Tape samples. **(B)** Double-thickness paper (S-S) samples.

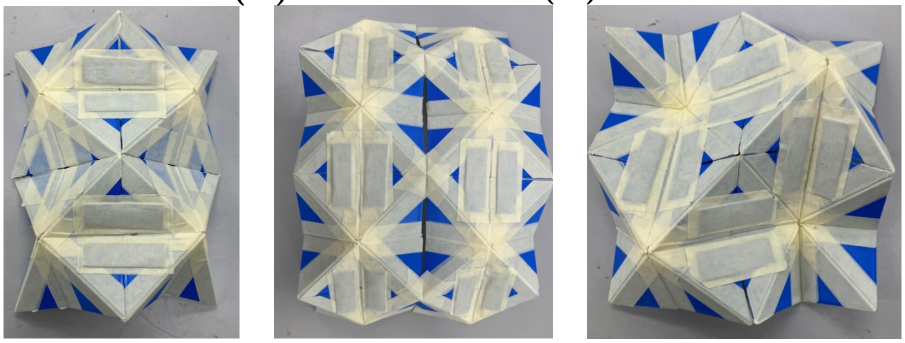

Fig. S6: 2D array models with different attachments. Three different attachment methods are explored. **(A)** In the first attachment method, the masking tape is used to attach the centre of each facet of the 1D arrays. **(B)** In the second attachment method, masking tape is used to attach the outer corner vertices of the facets of each unit in the 1D array. **(C)** In the third attachment method, the earlier two methods are merged simultaneously, where the masking tapes are used to attach the centre of each facet in both directions.

Table S3: Tensile speed and length for different tapes

Tape Material	Tensile Speed [mm/min]	Length [mm]
Masking Tape	12	200
High-Temperature Polyester Tape	30	200
Duct Tape	30	200
Kinesiology Tape	30	50

Table S4: Permanent magnets chosen and their specifications

Permanent Magnets	Material	Dimension [mm×mm]
Assemtech Magnet	Neodymium Iron Boron	$\varnothing 3 \times 1$
Eclipse Pot Magnet (a, b)	Samarium-Cobalt	(a) $\varnothing 6 \times 4.5$<br>(b) $\varnothing 10 \times 4.5$
N42 Magnet	Neodymium Iron Boron	$\varnothing 2 \times 1$
N35 Magnet (a, b)	Neodymium Iron Boron	(a) $\varnothing 3 \times 1.5$<br>(b) $\varnothing 5 \times 2$
N52 Magnet	Neodymium Iron Boron	$\varnothing 10 \times 5$

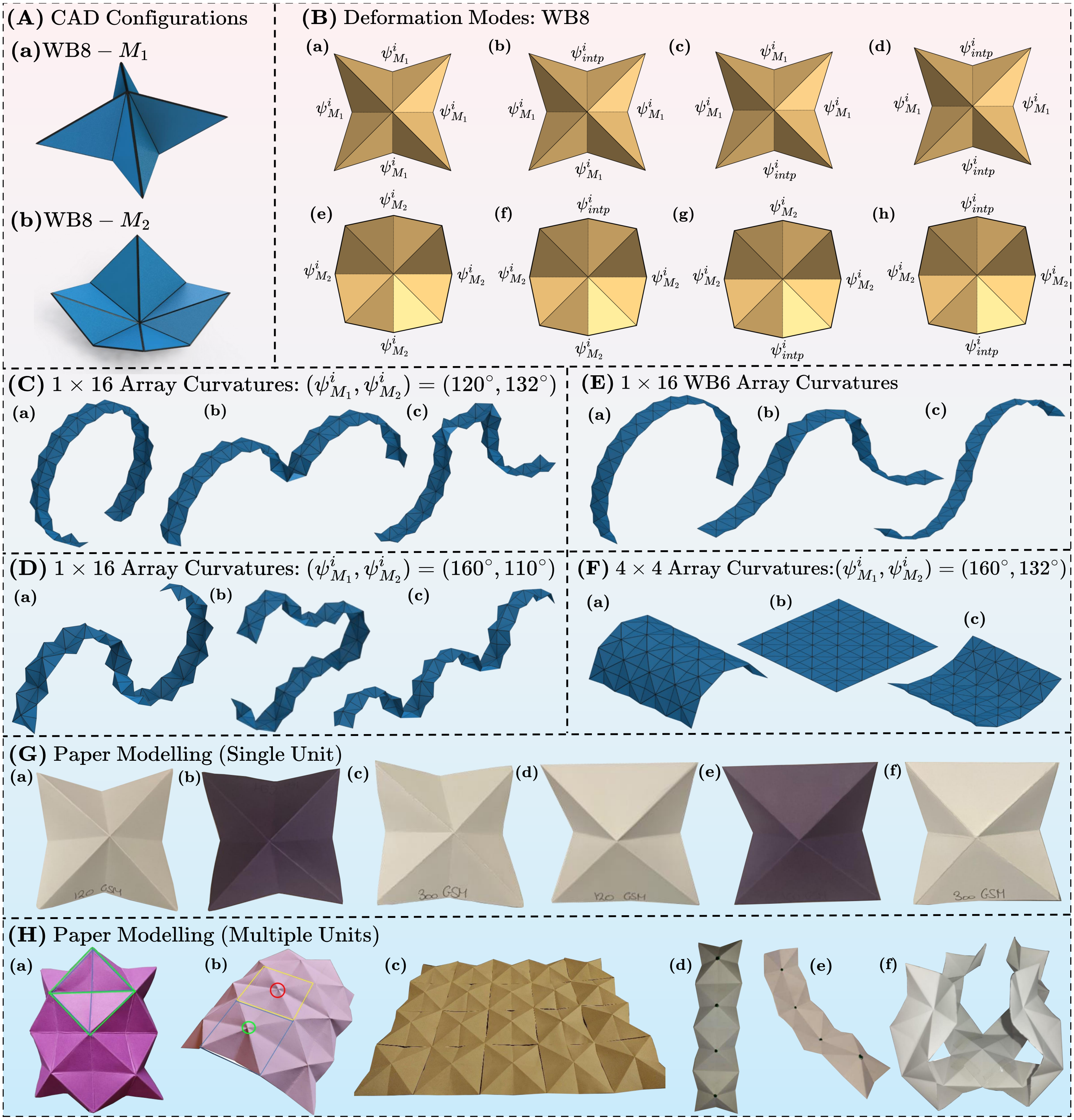

Fig. S7: Conceptual assembly of waterbomb units for achieving target metasurface curvatures. (A) Waterbomb unit model: (a) WB8-M_1 configuration, (b) WB8-M_2 configuration. **(B)** Deformation modes of a single WB8 in a 1D array: (a) M_1 connected to M_1 above and below, (b) M_1 connected to M_2 above and M_1 below, (c) M_1 connected to M_1 above and M_2 below, (d) M_1 connected to M_2 both above and below, (e) M_2 connected to M_2 above and below, (f) M_2 connected to M_1 above and M_2 below, (g) M_2 connected to M_2 above and M_1 below, (h) M_2 connected to M_1 both above and below. **(C - E)** Demonstration of achievable curvatures through locally programmable stability states in a 1 × 16 array with (C) WB8 units having $\psi^i_{M_1} = 120^\circ$ and $\psi^i_{M_2} = 132^\circ$ (D) WB8 units having $\psi^i_{M_1} = 160^\circ$ and $\psi^i_{M_2} = 110^\circ$. (E) WB6 units. **(F)** Demonstration of 2D curvatures achievable using 4×4 WB8 2D array with $\psi^i_{M_1} = 160^\circ$ and $\psi^i_{M_2} = 132^\circ$. **(G)** Waterbomb models with engineering-grade papers (single unit): (a) 120 gsm WB8, (b) 160 gsm WB8, (c) 300 gsm WB8, (d) 120 gsm WB6, (e) 160 gsm WB6, (f) 300 gsm WB6. **(H)** Waterbomb models with engineering-grade papers (multiple units): (a) Single folded paper forming a 2D array with blue creases and green in-phase plane facets, (b) 3 × 3 2D paper model with symmetrical boundary lines, (c) Flat plane of taped 2D array, (d) A strip of zero-stress 8-creases waterbomb array (globally flat), (e) Three out of four units are inverted to produce a curve configuration, (f) WB8 based gripper model that works through alteration of waterbomb unit stability states.

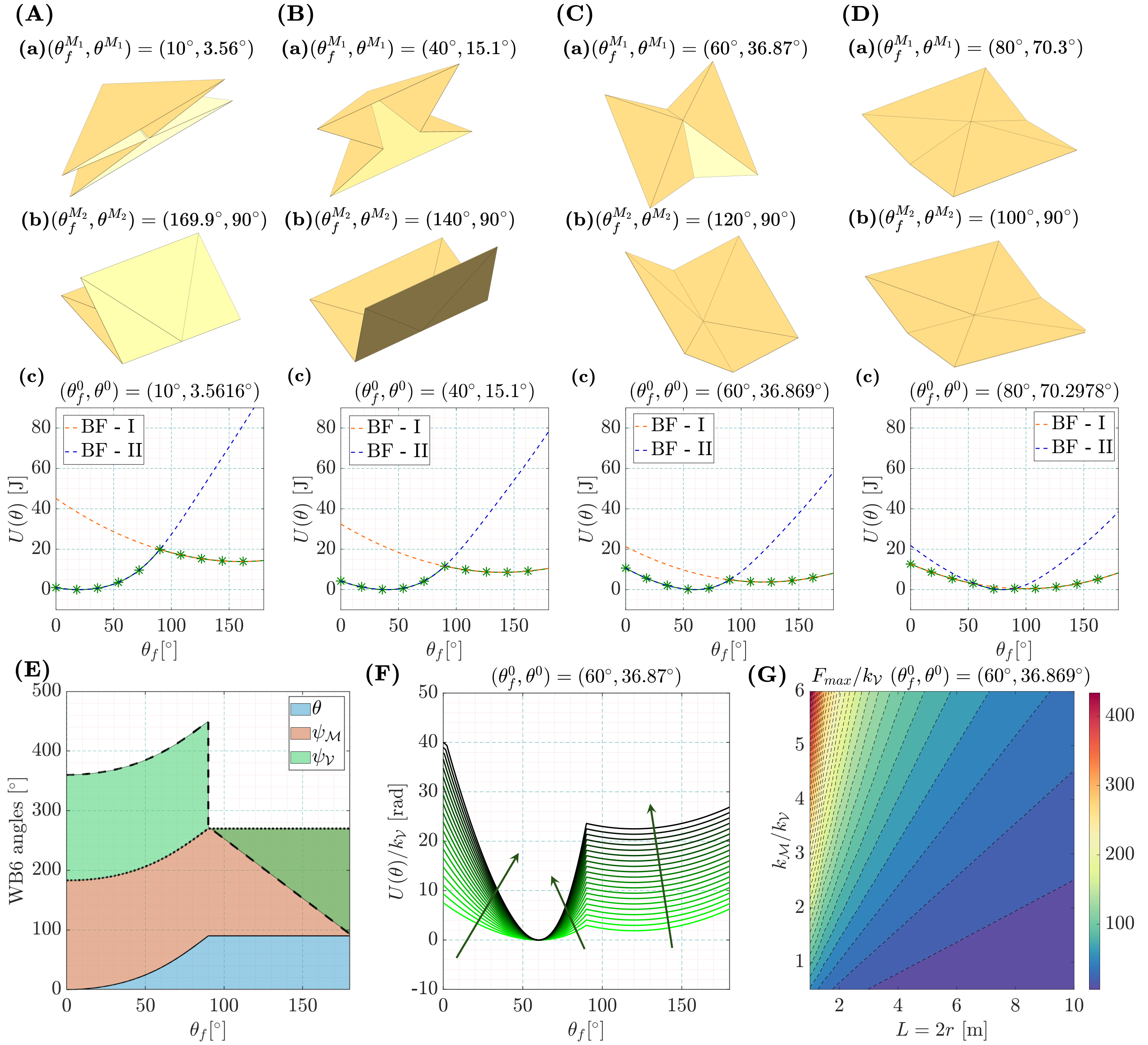

Fig. S8: Stability states of six-crease waterbomb units with rotational asymmetry. (A - D) The bi-stable configurations (M_1 and M_2) of the WB6 with their corresponding θ_f and θ are shown for the four initial rest angle cases. The bi-stable configurations are calculated based on the potential energy, considering two possible bifurcation motions [1]. The notation (✳) shows the path with the minimum potential energy, which is eventually followed by the structure. The four rest angles assumed here are as follows: (A) $\theta_f^0 = 10^\circ$ (B) $\theta_f^0 = 40^\circ$ (C) $\theta_f^0 = 60^\circ$ (D) $\theta_f^0 = 80^\circ$. **(E)** Variation of geometric angles of the waterbomb as a function of the given input angle θ_f. Here the geometric angles include the alternative input angle (θ), the dihedral angles corresponding to the mountain ($\psi_{\mathcal{M}}$) and valley ($\psi_{\mathcal{V}}$) crease. **(F)** Variation of the potential energy ($U(\theta)$) as a function of given angle θ_f for different stiffness ratio of waterbombs ($k_{\mathcal{M}}/k_{\mathcal{V}} = 1 \cdots 6$) with the initial rest angle (θ_f^0) of 60°. The arrow (⟶) highlights the increment direction of the stiffness-ratio. **(G)** Contour plot of the maximum inverting force, $F_{max} = F(\theta = \pi/2)$, required for the bi-stable shape transition as the function of the crease stiffness ratio and the dimension (side length, L) of the waterbomb square. The rest angle is set here at $\theta_f^0 = 60^\circ$ (or $\theta^0 = 36.869^\circ$).

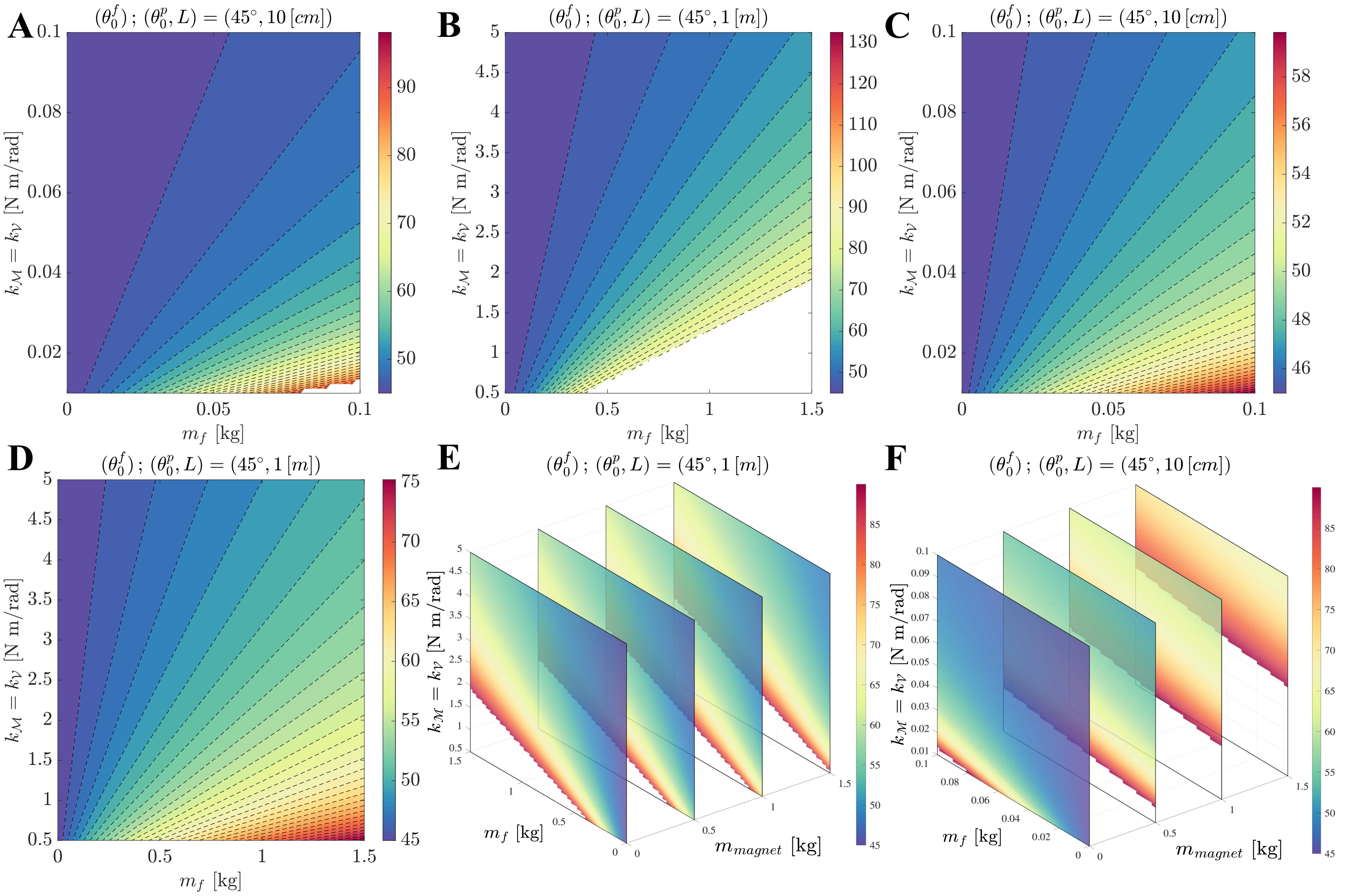

Fig. S9: Influence of facet mass on the initial natural rest angles of waterbomb units. Contour plots are presented for the final rest angle (θ_0^f) as a function of the crease stiffness ($k_{\mathcal{M}}$, $k_{\mathcal{V}}$) and the facet mass (m_f) in the waterbomb units with natural rest angle $\theta_0^p = 45°$ and the following parameters: **(A)** $(L, N) =$ (10cm, 8), **(B)** $(L, N) =$ (1m, 8), **(C)** $(L, N) =$ (10cm, 6), **(D)** $(L, N) =$ (1m, 6). L represents the side length of a square waterbomb unit , while N denotes the number of folds within it. Note that crease stiffness for both the valley and mountain folds is taken as the same here i.e., $k_{\mathcal{M}} = k_{\mathcal{V}}$. The influence of attaching permanent tip-magnet is shown for the waterbomb unit of $N = 8$ with **(E)** $L = 1m$ and **(F)** $L = 10cm$.

Table S5: Test results for attraction (F_a) and repulsion (F_r) force for 4 magnets at different air gap distances

Magnet Types		Air gap distance						
		10[mm]	15[mm]	20[mm]	25[mm]	30[mm]	35[mm]	40[mm]
Eclipse Pot Magnet (a)	F_a[N]	n/a						
	F_r[N]	-0.0086	-0.0015	-0.0004	-0.0004	n/a		
Eclipse Pot Magnet (b)	F_a[N]	n/a						
	F_r[N]	-0.0807	-0.2680	-0.0107	-0.0063	n/a		
N35 Magnet (b)	F_a[N]	0.0412	0.0215	0.0088	0.0038	n/a		
	F_r[N]	-0.0315	-0.0113	0.0062	-0.0006	n/a		
N52 Magnet	F_a[N]	1.4230	0.5640	0.0283	0.1738	0.1085	0.5880	0.0377
	F_r[N]	-1.2700	-0.4940	-0.2655	-0.1440	-0.7010	-0.0397	-0.0265

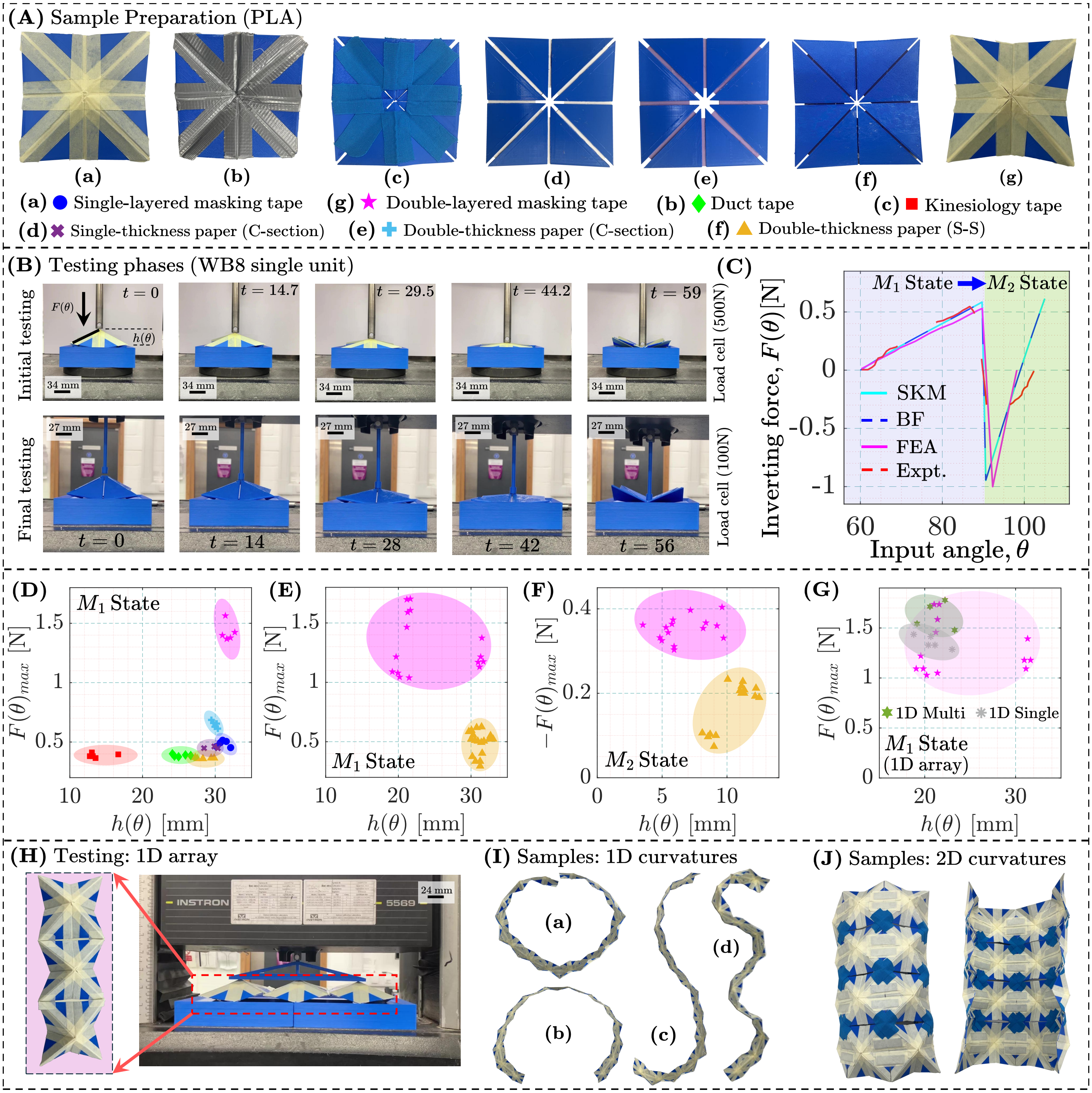

Fig. S10: Experimental investigation of the structural properties of waterbomb assemblies. **(A)** WB8 Single unit test samples with 3D printed PLA facets and attachment methods : **(a)** Single-layered masking tape, **(b)** Duct tape, **(c)** Kinesiology tape, **(d)** Single-thickness paper (C-section), **(e)** Double-thickness paper (C-section), **(f)** Double-thickness paper (S-S), **(g)** Double-layered masking tape. **(B)** Force-displacement testing set-up for the single unit using two different crease materials, presented in the top (initial testing using 500N load cell) and bottom (final testing, narrowed down to 100N load cell) rows, respectively. **(C)** Experiment and finite-element based validation of WB8's inverting force profile with analytical models from literature, SKM [7] and BF [1]. **(D)** Inverting force (F_{max}) vs vertex ($h = r\cos\theta$) height with WB8-M_1 crease material samples (initial testing, using 500N load cell). **(E)** Inverting force (F_{max}) vs vertex ($h = r\cos\theta$) height with selected WB8-M_1 crease material samples (final testing, narrowed down to 100N load cell). **(F)** Inverting force (F_{max}) vs vertex ($h = r\cos\theta$) height with selected WB8-M_2 crease material samples (final testing, narrowed down to 100N load cell). **(G)** Inverting force (F_{max}) vs vertex ($h = r\cos\theta$) height: 1×3 array sample testing. **(H)** 3D printed 1D array testing set-up with 1×3 WB array. **(I)** Physical prototypes of 1×14 WB8 array, demonstrating the ability of curvature variation. **(J)** Physical prototypes of 4×4 WB8 array, demonstrating the ability of curvature variation.

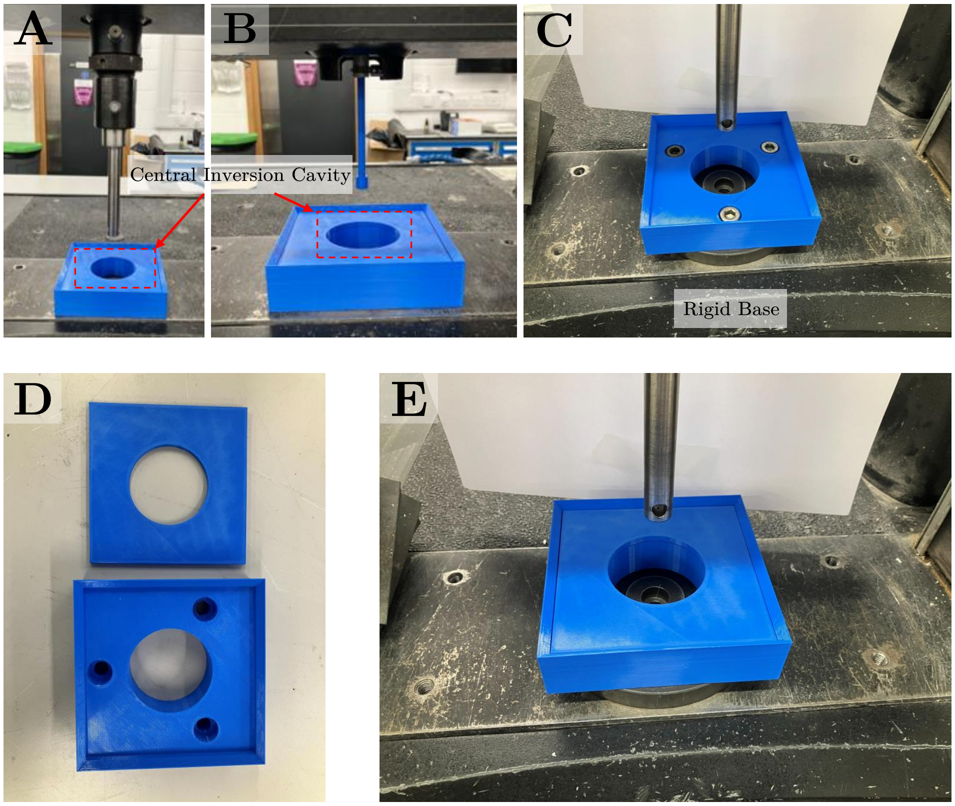

Fig. S11: Rigid jig designs for testing of waterbomb inversion. It shows a central hollow cavity which is to accommodate the sample after its inversion to second inverted state and also to restrain movement of the sample during testing into undesired directions.

Table S6: Test results for magnetic actuator

Air Gap Distance [mm]	Attraction Force [N]	Repulsion Force [N]
27	1.128	-0.7166
32	0.7407	-0.4400
37	0.3698	-0.2746
42	0.2186	-0.1465
47	0.1512	-0.0845
52	0.0966	-0.0526
57	0.0572	-0.039
62	0.0345	-0.0304

References

[1] H. Zhang, B. Zhu, B. Chen, C. Cui, H. Li, X. Zhang, A phase diagram-based stability design method for a symmetrical origami waterbomb base, Journal of Mechanical Design 144 (2022) 103303.

[2] M. Grasinger, A. Gillman, P. R. Buskohl, Multistability, symmetry and geometric conservation in eightfold waterbomb origami, Proceedings of the Royal Society A 478 (2022) 20220270.

[3] Z. Qi, X. Sun, J. Xu, A kirigami multi-stable flexible gripper with energy-free configurations switching, Advanced Intelligent Systems 6 (2024) 2400038.

[4] C. Liu, P. Maiolino, Z. You, A 3d-printable robotic gripper based on thick panel origami, Frontiers in Robotics and AI 8 (2021) 730227.

[5] A. Mathew, C. Rao, A cad system for extraction of mating features in an assembly, Assembly Automation 30 (2010) 142–146.

[6] S. Tickoo, SOLIDWORKS 2020 for Designers, Cadcim Technologies, 2020.

[7] B. H. Hanna, Modeling and testing of bistable waterbomb base configurations, Brigham Young University, 2014.
